# Stacked enviromic–genomic models improve prediction of genotype performance in new environments

**DOI:** 10.1093/g3journal/jkag257

**Published:** 2026-09-18

**Authors:** Marcos Antonio de Godoy Filho, Mauricio dos Santos Araújo, José Tiago Barroso Chagas, José Baldin Pinheiro

**Affiliations:** Department of Genetics, Genetics Diversity and Breeding Laboratory, University of São Paulo, Piracicaba, São Paulo,13418-900, Brazil; Department of Genetics, Genetics Diversity and Breeding Laboratory, University of São Paulo, Piracicaba, São Paulo,13418-900, Brazil; Institute of Agricultural Sciences, Federal University of Jataí (UFJ), Jataí, Goiás, 75801-615, Brazil; Department of Genetics, Genetics Diversity and Breeding Laboratory, University of São Paulo, Piracicaba, São Paulo,13418-900, Brazil; Department of Genetics, Genetics Diversity and Breeding Laboratory, University of São Paulo, Piracicaba, São Paulo,13418-900, Brazil

**Keywords:** phenotypic plasticity, extreme gradient boosting, factor analytic model, manifold learning (PaCMAP), reaction norms, genomic prediction

## Abstract

Genotype-by-environment interaction is a major challenge for breeding programs, limiting the predictive ability of genomic selection in untested environments. We propose a Stacked Generalization framework that integrates linear mixed models (factor analytic and genomic best linear unbiased prediction), enviromic reaction norms, and machine learning (Extreme Gradient Boosting) to predict phenotypic plasticity. The framework was evaluated on large multi-environment trials of maize and rice, under scenarios that simulate new environments and seasons. The genetic covariance structures differed between crops, requiring a factor analytic model of order k = 6 for continental maize and order k = 2 for the local rice network. Across all scenarios, the Stacking ensemble improved on the genomic baseline (M1), with gains in predictive ability from 10% (r=0.45 vs. 0.41 for M1) to 27.5% (r=0.51 vs. 0.40), and reduced the root mean squared error by 30% to 43% relative to the Enviromic Reaction Norm (M2) when ensembles were selected to minimize error. These gains relied on careful feature engineering. Latent variables from genomic and environmental dimensionality reduction (principal component analysis and PaCMAP) and their interactions were the most important features for the machine learning models, and the first genomic PaCMAP component ranked first for both crops. These results indicate that non-linear dimensionality reduction is a promising tool for genomic and enviromic prediction. By combining the stability of mixed models with the flexibility of machine learning, the framework improves robustness, reduces dependence on any single model, and enhances prediction in new environments, supporting cultivation zone expansion and recommending superior genotypes.

## Teaser

Plant breeders need to predict how crop varieties will perform in locations and seasons they have never been tested. Current methods often fail because they rely on simplified models that miss complex responses to climate variation. The authors developed a framework that combines traditional statistical models with machine learning, integrating genomic and environmental data from maize and rice trials. Their approach improved prediction accuracy by up to 27.5% compared to standard methods. By tracing the algorithm's decisions back to biological drivers, they confirmed the models learned real physiological responses, supporting better crop recommendations and adaptation strategies.

## Introduction

Genotype-by-environment (G×E) interaction represents one of the major challenges for the efficiency of breeding programs, as the differential response of genotypes to environmental variation hinders the selection of stable and broadly adapted genotypes (Cooper and DeLacy 1994; Araújo et al. 2024). Traditionally, the analysis of multi-environment trials (MET) relied on linear regression models or multivariate analyses of variance, such as the additive main effects and multiplicative interaction (AMMI) model, to describe G×E patterns (Finlay and Wilkinson 1963; Gauch 1988). However, such approaches are primarily descriptive and present limitations in predicting the performance of untested genotypes or in unobserved environments.

The implementation of genomic prediction (GP) established a new paradigm by enabling the prediction of breeding values based on dense molecular markers, thereby accelerating genetic gain per unit of time (Bernardo 1994; Meuwissen et al. 2001). The Genomic Best Linear Unbiased Predictor (GBLUP) became the benchmark due to its statistical robustness and computational efficiency. Nevertheless, its conventional application relies on the genomic relationship matrix proposed by VanRaden (2008), which strictly models linear and additive marker effects. This framework restricts the capture of complex non-additive genetic effects, such as dominance and epistasis, which require alternative relationship matrices (Vitezica et al. 2013). Furthermore, because it relies primarily on genomic and phenotypic information, its ability to explicitly model reaction norms and G×E responses to climatic variation remains limited (Jarquín et al. 2014).

To mitigate the limitations of purely genomic models, the concept of Enviromics emerged, proposing the integration of high-dimensional environmental covariates (climate, soil, and management) into prediction models (Xu 2016; Costa-Neto, Fritsche-Neto et al. 2021; Resende et al. 2021, 2024). Direct modeling of environmental features interacting with genomic covariance through the Hadamard product has demonstrated significant gains in predictive ability by capturing reaction norms in a continuous manner, as originally proposed by Jarquín et al. (2014).

Parallel to the explicit integration of environmental covariates, accurate modeling of the between-environment genetic covariance structure remains a fundamental pillar for improving predictive ability. Factor Analytic (FA) models, originally detailed by Piepho (1997) and Smith A et al. (2001), remain state-of-the-art for parsimoniously modeling latent genetic correlations across environments (Araújo et al. 2025; Hrachov et al. 2026). Studies such as Araújo et al. (2024) have expanded this methodology by integrating latent factor loadings with Geographic Information Systems (GIS) and envirotyping data. This modern fusion not only accommodates the imbalance typical of MET data but also refines the characterization of phenotypic stability, supports the definition of adaptation zones, and enables extrapolative prediction of genotype performance in untested environments.

Concurrently with advances in linear mixed models, non-linear kernel methods have been explored to capture non-additive genetic patterns and complex quantitative trait architectures across diverse crops and environmental conditions. The Arc-Cosine kernel mimics the representational capacity of deep neural networks within a kernel framework, enabling the modeling of epistatic interactions and non-linear patterns without the computational burden of dense neural architectures (Cuevas et al. 2019). Costa-Neto, Fritsche-Neto et al. (2021) validated the application of such kernels under enviromic scenarios, demonstrating superiority over linear GBLUP in the presence of dominance effects and complex G×E interaction.

Despite the robustness of statistical and kernel-based methods, machine learning (ML) algorithms such as gradient boosting and neural networks have demonstrated high capability in capturing complex and non-linear patterns in large datasets of maize, wheat, rice, and groundnut (Montesinos-López OA et al. 2018; Fernandes et al. 2024; Montesinos-López A et al. 2025). These studies indicate that ML and deep learning models may outperform GBLUP when modeling intricate genetic architectures and strong G×E interactions, while emphasizing the importance of careful regularization and hyperparameter tuning to ensure generalization (Montesinos-López A et al. 2025).

A methodology currently gaining prominence in genomic prediction is the integration of multiple approaches through Ensemble Learning and Stacked Generalization. Yu et al. (2025) introduced the EXGEP framework, demonstrating that combining diverse base models (e.g. LightGBM and random forest) via a meta-model consistently improves predictive performance relative to individual models. The rationale behind stacking is that different algorithms can capture complementary variance components: statistical mixed models represent global covariance structures, whereas boosting-based methods capture local and non-linear interactions.

Beyond algorithm selection, feature engineering and dimensionality reduction are critical for enhancing ML performance in high-dimensional genomic and environmental contexts. Resende et al. (2025) introduced the concept of enviromic marker engineering, using random forest algorithms to transform raw environmental data into synthetic predictors that maximize explanatory power. In parallel, non-linear dimensionality reduction techniques from Manifold Learning, such as Pairwise Controlled Manifold Approximation (PaCMAP), have gained prominence for overcoming the linear limitations of Principal Component Analysis (PCA) by preserving both global and local data structures (Wang et al. 2021). Recently, Godoy Filho et al. (2026b) demonstrated the effectiveness of integrating PaCMAP components into genetic models to classify environments and dissect crossover G×E interaction in maize. The incorporation of these non-linear embeddings enables tree-based algorithms to navigate complex G×E search spaces more effectively.

In this study, we evaluate a systematic genomic prediction framework that explores the synergy between classical linear mixed model inference (FA and GBLUP) and the predictive flexibility of Ensemble Learning. We propose a stacked generalization architecture designed to integrate models capturing distinct biological signals, combining global additive structure with sensitivity to local non-linear interactions. The objective is to validate the efficacy of these hybrid configurations for predicting genotype performance in unobserved environments, using maize and rice datasets to demonstrate the framework’s ability to generalize complex G×E patterns. Ultimately, this approach supports strategies for cultivation zone expansion and the recommendation of superior genotypes.

## Materials and methods

### Phenotypic data and first-stage analysis—maize

The phenotypic data were sourced from the Genomes to Fields (G2F) (Chen Q et al. 2026). The analyzed dataset comprised a set of 183 maize hybrids evaluated across 180 unique environments covering a wide latitudinal range across the United States and Ontario, Canada (Fig. 1). An environment was defined as a unique combination of location and year. Consequently, distinct trials conducted within the same site were jointly analyzed as a single environment, given that most trials were planted on nearly the same dates. The trials were conducted using predominantly randomized complete block designs (RCBD) or incomplete block designs, typically with two replications.

**Fig. 1.**
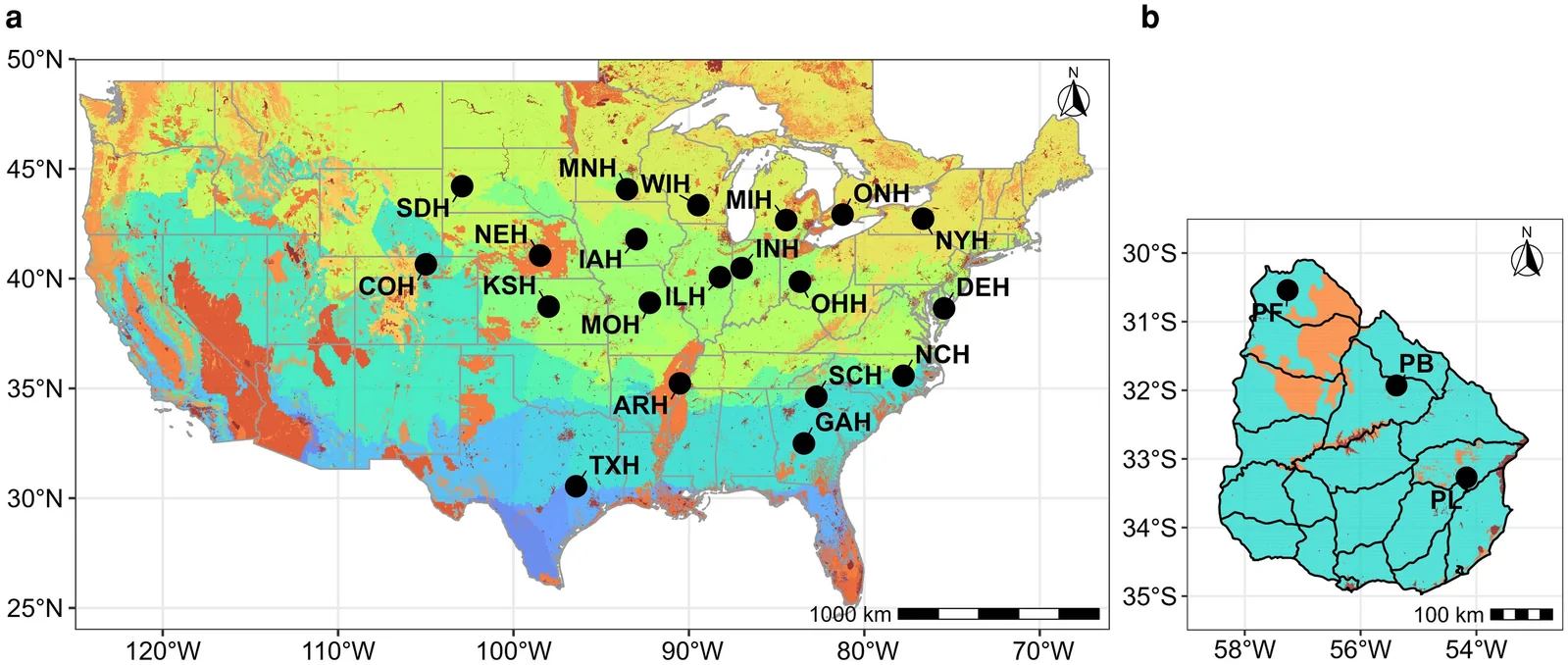
Geographic distribution of trials overlaid on the Agroecological Zones of North America (FAO 2025). Black dots represent the locations of the trials a) United States and Canada (e.g. site ONH in Ontario). The background colors indicate distinct Agroecological Zones (AEZs), illustrating the vast climatic and environmental diversity sampled. b) Uruguay map, the background colors also indicate the AEZ. highlighting the stark differences in geographic dispersion and territorial extent between the two datasets. This spatial contrast ranging from the continental scale of the US/Canada trials to the more localized distribution in Uruguay is a driver of the observed G×E interaction complexities (estimated by FA model) in each unique dataset.

A two-stage analysis was performed, where environments were first analyzed individually to obtain the Best Linear Unbiased Estimator (BLUEs). To accommodate the diversity of experimental designs and the presence of environments containing multiple trials, the model structure was dynamically adjusted using the *ASReml-R* package (Butler et al. 2017). The general linear mixed model fitted was:


(1) $$y=X\beta +Z_{b}u_{b}+\epsilon$$

where y is the n×1 vector of observed phenotypic values (grain yield), with *n* being the total number of plots in the environment. β is the p×1 vector of fixed effects, including the intercept, genotype, replication, and trial effects (when applicable). X is the n×p design matrix relating observations to fixed effects. $u_{b}$ is the q×1 vector of random block effects, nested within trials and replications in incomplete block designs, assumed as $u_{b}∼N(0,I_{q}\sigma_{b}^{2})$  $I_{q}$ is an identity matrix of order *q*. $Z_{b}$ is the n×q design matrix for random block effects.

To account for the diversity of experimental designs and potential heteroscedasticity, the model structure was dynamically adjusted. For environments containing multiple trials, a fixed trial effect was included, and a heterogeneous residual variance structure was modeled as ϵ∼N(0,R), where $R=\overset{t}{\underset{k=1}{⨁}}I_{n_{k}}\sigma_{e_{k}}^{2}$, with *t* representing the number of trials and $n_{k}$ the number of plots in the *k*th trial.

To assess trial quality, the generalized heritability ($H_{\mathrm{Cullis}}^{2}$) was estimated according to Cullis et al. (2006). For this purpose, models were re-fitted considering genotype effects as random, $g∼N(0,I\sigma_{g}^{2})$. The generalized heritability was calculated as:


(2) $$H_{\mathrm{Cullis}}^{2}=1-\frac{{\bar{v}}_{\Delta \mathrm{BLUP}}}{2\sigma_{g}^{2}}$$

where ${\bar{v}}_{\Delta \mathrm{BLUP}}$ is the mean variance of the difference between two BLUPs and $\sigma_{g}^{2}$ is the genotypic variance.

To predict the performance of known genotypes in unobserved environments and years, we retained only hybrids evaluated in at least 50 environments.

The experimental coefficient of variation ($CV_{e}$) was calculated as:


(3) $$CV_{e}=\frac{\sqrt{\sigma_{e_{k}}^{2}}}{\bar{Y}}$$

where $\sigma_{e_{k}}^{2}$ is the residual variance for the *k*th trial and $\bar{Y}$ is the mean grain yield.

### Phenotypic data and first-stage analysis—rice

The rice dataset was obtained from the breeding program of the National Agricultural Research Institute (INIA) of Uruguay (Rebollo et al. 2023). The analyzed dataset comprised a set of 73 breeding lines and 69 environments. The germplasm consisted of elite breeding lines belonging to the *indica* and *japonica* subspecies of *O. sativa*. The trials were conducted under irrigated conditions across three locations in Uruguay: Paso Farías (PF), Pueblo del Barro (PB), and Paso de la Laguna (PL) (Fig. 1b). This experimental network is geographically compact, spanning a latitudinal and longitudinal range of less than 3 degrees (approximately 300 km). This stands in stark contrast to the maize dataset, which covers a continental scale with massive photoperiodic and thermal variations. Consequently, the rice trials constitute a spatially homogeneous network where the primary sources of environmental variation are temporal (years) and management-driven (sowing dates).

To maximize the robustness of environmental characterization, trials were aggregated into environments based on a dynamic temporal clustering approach nested within Year and Location. Sowing dates were sorted chronologically, and a new environmental cluster was defined whenever the temporal distance to the first sowing date of the current cluster exceeded a threshold of 10 days. This ensured that trials with similar photothermal regimes were analyzed jointly, while distinct sowing epochs were treated as separate environments.

The same two stage strategy was applied for the rice dataset, where environments were first analyzed individually to obtain BLUEs. The experimental design followed a RCBD with two to four replications. To account for potential heterogeneity among trials constituting an environment, the following linear mixed model was fitted:


(4) $$y=X\beta +Z_{r}u_{r}+\epsilon$$

where y is the n×1 vector of observed phenotypic values (grain yield). β is the p×1 vector of fixed effects, including the intercept, genotype, and trial effects. $Z_{r}$ is the n×q design matrix for random effects, and $u_{r}$ is the q×1 vector of random replication effects nested within trials. The random replication effects were assumed to be $u_{r}∼N(0,I_{q}\sigma_{r}^{2})$. To account for potential heteroscedasticity, a heterogeneous residual variance structure was modeled for each trial within the environment ϵ∼N(0,R), $R=\overset{t}{\underset{k=1}{⨁}}I_{n_{k}}\sigma_{e_{k}}^{2}$, with *t* representing the number of trials and $n_{k}$ the number of observations in the *k*th trial.

Trial quality was assessed using the generalized heritability ($H_{\mathrm{Cullis}}^{2}$), estimated by re-fitting the model considering genotype as a random effect $g∼N(0,I\sigma_{g}^{2})$. The same coverage criterion was applied to the lines, retaining those evaluated in at least 10 environments; the lower threshold reflects the smaller number of environments in the rice network (69 vs. 180).

### Environmental characterization and dimensionality reduction

To capture environmental diversity and mitigate the high dimensionality of climatic data, a unified framework was applied to both maize and rice datasets using the NASA POWER database (Sparks 2018). The *EnvRtype* package (Costa-Neto, Galli et al. 2021) was employed to process daily meteorological data, computing 33 environmental covariates (Supplementary Table S1). The mean of these features was aggregated within specific phenological windows tailored to the developmental biology of each crop.

For rice, in environments where days to flowering were unavailable, values were imputed using the historical mean observed for the corresponding sowing date and region. Harvest dates were estimated as 30 days post-flowering (Yoshida 1981). Given the limited latitudinal variation in the Uruguayan trials, the rice cycle was divided into four fixed phenological windows based on accumulated Growing Degree Days (GDD), following developmental stages described by Counce et al. (2000) and Yoshida (1981). W1 (0–300 GDD) covers the seedling and establishment phase (Stages S0–S3), critical for stand survival. W2 (300–900 GDD) encompasses active tillering and the vegetative phase, capturing the plasticity of leaf area and tiller formation. W3 (900 GDD to flowering) corresponds to the reproductive phase (Panicle Initiation to Anthesis), where yield potential is largely determined. W4 (flowering to harvest; approximately 440 GDD) represents the grain filling and maturation process.

In contrast to rice in Uruguay, where environmental homogeneity permits the use of fixed thermal windows, such an approach is unfeasible for maize across the United States. Non-linear responses to supra-optimal temperatures (Kumudini et al. 2014) and photoperiod sensitivity (Drouault et al. 2025) significantly alter the thermal duration of the cycle, causing absolute GDD values to represent distinct phenological stages across different latitudes. To address this, the maize vegetative phase was segmented into three windows based on the percentage of total GDD accumulated from planting to silking: W1 (0–40%), W2 (40–90%), and W3 (90–100%). Similarly, the reproductive phase was stratified into four windows based on GDD accumulation from silking to harvest: W4 (0–10%), W5 (10–30%), W6 (30–65%), and W7 (65–100%) (Godoy Filho et al. 2026b).

Following feature aggregation, the resulting phenological feature matrix $X_{\mathrm{EnvPheno}}$ underwent dimensionality reduction to capture similarity structures among environments. The Pairwise Controlled Manifold Approximation (PaCMAP) technique (Wang et al. 2021) was applied with hyperparameters set to n_neighbors=6, MN_ratio=0.5, and FP_ratio=4, yielding three components to capture local non-linear structures. These were combined with the first ten principal components derived from a PCA, constituting the final latent environmental descriptor matrix $X_{\mathrm{EnvLatent}}$, totaling 13 features.

### Genomic data processing and interaction modeling

Genomic data acquisition respected the specific genotyping platforms of each crop, while quality control (QC) and downstream modeling followed a shared protocol to ensure methodological consistency. In maize, hybrid genotypic data were obtained from the 2024 G×E Interaction Prediction Competition (Chen Q et al. 2026). Raw data were generated by the G2F initiative, involving parental line sequencing, variant calling using the Practical Haplotype Graph (PHG), and *in silico* projection of hybrid genotypes through TASSEL 5.0. The dataset comprised 2,425 SNP markers. For rice, genomic data were obtained as described by Rebollo et al. (2023). Genotyping was performed via Genotyping-by-Sequencing (GBS) using the *ApeKI* restriction enzyme. Raw sequence processing and variant calling were conducted using the TASSEL 5.0 pipeline, aligning SNPs against the *Nipponbare* reference genome (*MSU* version 7.0). The consolidated rice genomic matrix contained approximately 65,000 markers.

### Unified processing and feature engineering

A rigorous QC procedure was applied to both datasets: markers with a missing data rate greater than 10% or minor allele frequency (MAF) lower than 3% were removed, and remaining missing values were imputed using the marker mean. The filtered genomic matrices were subsequently subjected to dimensionality reduction to generate latent genomic descriptor matrices, denoted as $X_{\mathrm{GenLatent}}$. Two complementary approaches were employed: (i) Principal Component Analysis (PCA), retaining the first 10 components to capture global linear genomic structure; and (ii) Genomic PaCMAP, extracting three components using the same hyperparameters adopted in the environmental step, to capture local non-linear genomic relationships.

Finally, to engineer explicit interaction terms, a structured interaction matrix $X_{G\times E}$ was constructed. Each of the six genomic axes (three PCA and three PaCMAP components) was multiplied element-wise (Hadamard product, $g_{i}⊙e_{j}$) by each of the six environmental axes (three PCA and three PaCMAP), producing the 36 interaction terms in $X_{G\times E}$. This strategy enables explicit modeling of phenotypic plasticity by capturing interaction signals between latent genomic and environmental drivers while avoiding excessive dimensionality inflation.

### Predictive strategies (M1–M6)

To systematically evaluate the integration of enviromic and genomic signals, six predictive strategies (M1 to M6) were implemented and categorized into three modeling frameworks: (i) Baseline models, (ii) Feature-based Machine Learning, and (iii) Target-oriented learning (Fig. 2).

**Fig. 2.**
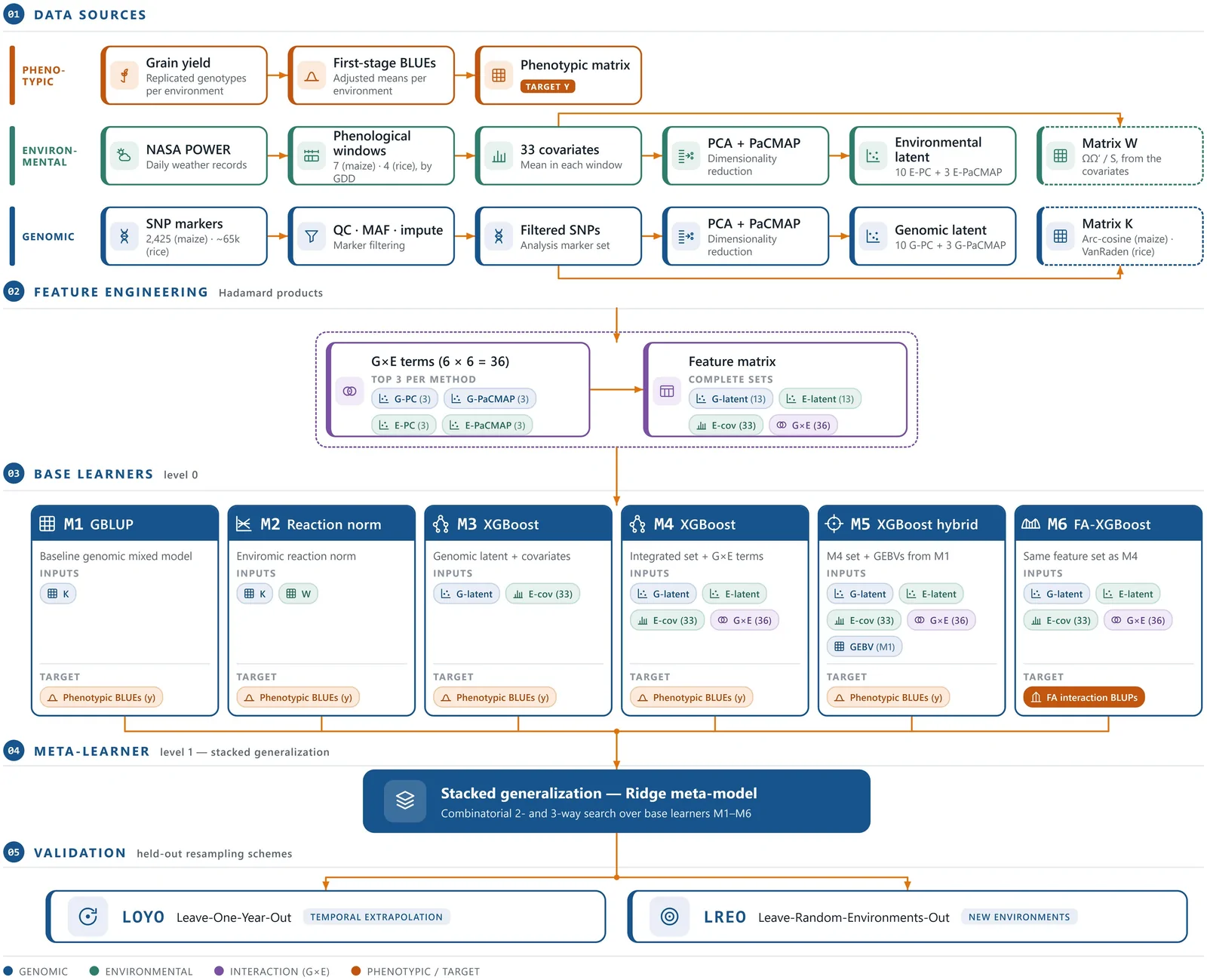
Analytical pipeline of the stacked enviromic–genomic framework, shown as five sequential stages. **(01) Data sources:** phenotypic (grain-yield BLUEs), environmental (33 covariates aggregated in phenological windows), and genomic (SNP markers) inputs, each reduced by PCA and PaCMAP into latent descriptors. **(02) Feature engineering:** the feature matrix combines the latent descriptors with explicit G×E interaction terms, obtained as the element-wise (Hadamard) products of the top three genomic and top three environmental components. **(03) Base learners (Level 0):** M1-GBLUP (baseline linear mixed model) and M2-Reaction Norm (enviromic reaction norm), plus four XGBoost strategies: M3 (latent genomic descriptors with phenological environmental features), M4 (M3 plus the explicit interaction terms), M5 (M4 plus genomic estimated breeding values from M1), and M6 (same features as M4 but trained to predict the interaction BLUPs of a factor analytic model). **(04) Meta-learner (Level 1):** a Ridge-regression stacked generalization combines the base-model predictions. **(05) Validation:** LOYO and LREO.

### Baseline models

To establish a benchmark for predictive performance and capture global additive and non-additive genetic signals, two baseline models (M1 and M2) were implemented. These models serve as statistical references against which machine learning and ensemble strategies are evaluated. Both models were fitted using the Bayesian Generalized Linear Regression (BGLR) *R* package (Pérez and de los Campos 2014). The Gibbs sampler was configured with 20,000 iterations, a burn-in period of 3,000, and a thinning interval of 5.

#### Model M1—genomic best linear unbiased predictor

This model accounts for the main genetic effects by modeling covariance among genotypes. The general linear mixed model is defined as:


(5) $$y=X\beta +Z_{g}u_{g}+\epsilon$$

where y is the n×1 vector of BLUE records; β is the p×1 vector of fixed effects including the intercept and environmental main effects; $u_{g}$ is the q×1 vector of random genotypic effects assumed as $u_{g}∼N(0,K\sigma_{g}^{2})$, where K is the q×q genomic relationship matrix and $\sigma_{g}^{2}$ is the genomic variance. X and $Z_{g}$ are incidence matrices of dimensions n×p and n×q, respectively. The residual term is defined as $\epsilon ∼N(0,I_{n}\sigma_{e}^{2})$ where $I_{n}$ is an identity matrix of order *n*.

To accommodate distinct genetic architectures across crops, the genomic relationship matrix K was specified as follows:

- For rice (inbred lines), the canonical additive genomic relationship matrix G proposed by VanRaden (2008) was used, capturing additive genetic variance expected in homozygous materials.
- For maize (hybrids), the Arc-Cosine kernel $K_{\mathrm{Arc}}$ was employed. Unlike linear kernels, $K_{\mathrm{Arc}}$ maps marker data into a high-dimensional feature space approximating an infinitely wide neural network, enabling the capture of dominance and epistatic effects prevalent in hybrid combinations (Cuevas et al. 2019).

#### Model M2—enviromic reaction norm

To explicitly model phenotypic plasticity and G×E interaction, M1 was extended through a reaction norm framework incorporating high-dimensional environmental covariates (Jarquín et al. 2014). The model is defined as:


(6) $$y=X\beta +Z_{g}u_{g}+Z_{e}u_{e}+Z_{ge}u_{ge}+\epsilon$$

where $u_{g}$ is the q×1 vector of main genotypic effects associated with $Z_{g}$ (n×q), $u_{e}$ is the J×1 vector of environmental main effects associated with $Z_{e}$ (n×J), and $u_{ge}$ is the qJ×1 vector of genotype-by-environment interaction effects associated with $Z_{ge}$ (n×qJ). The residual term is $\epsilon ∼N(0,I_{n}\sigma_{\epsilon }^{2})$. The environmental relationship matrix was computed as $W=\frac{\Omega \Omega^{⊤}}{S}$ where Ω represents the centered and standardized environmental covariate matrix and *S* is the number of covariates.

The variance–covariance structures were defined as $K_{G}=Z_{g}KZ_{g}^{⊤}$ and $K_{E}=Z_{e}WZ_{e}^{⊤}$. The interaction effects were modeled using a composite covariance structure based on the Hadamard product $u_{ge}∼N(0,[K_{G}⊙K_{E}]\sigma_{ge}^{2})$. This covariance formulation enables information borrowing across environmentally similar locations, thereby supporting extrapolative prediction in unobserved environments or growing seasons.

### Feature-based machine learning models

To capture non-linear patterns and complex high-order interactions that linear kernels may fail to detect, Extreme Gradient Boosting (XGBoost) models were implemented (Chen T and Guestrin 2016). In this framework, the target variable remained the phenotypic observations (BLUEs) for all models in this category. Predictor features were organized into an input matrix denoted as $X_{\mathrm{in}}$, with dimensions n×p, where *n* corresponds to the number of phenotypic observations and *p* represents the total number of predictors, varying according to the biological information included in each strategy.

To ensure robust convergence and mitigate overfitting, hyperparameters were optimized via grid search within each cross-validation fold. The search space included maximum tree depth (max_depth∈{4,5,6}), minimum child weight (min_child_weight∈{5,10,15}), and regularization parameters L1 (α∈{5.0,10.0,20.0}) and L2 (λ∈{1.0,10.0}). The learning rate was fixed at η=0.02 to prioritize generalization, and stochastic subsampling was applied by setting both row (subsample) and column (colsample_bytree) fractions to 0.75.

Model M3 represents a pure machine learning strategy relying on engineered biological features without explicit interaction terms. To enhance interpretability and physiological coherence, the input matrix was defined as:


(7) $$X_{\mathrm{in}}=\left[X_{\mathrm{GenLatent}}|X_{\mathrm{EnvPheno}}\right]$$

Model M4 expands this feature space by incorporating latent environmental descriptors and their explicit interactions with genomic features. Although boosting trees can implicitly learn interactions, explicit feature engineering accelerates convergence and stabilizes optimization. The full-spectrum input matrix is defined as:


(8) $$X_{\mathrm{in}}=\left[X_{\mathrm{GenLatent}}|X_{\mathrm{EnvPheno}}|X_{\mathrm{EnvLatent}}|X_{G\times E}\right]$$

Model M5 introduces biological priors into the learning process by augmenting the M4 feature space with genomic estimated breeding values (GEBVs) derived from the linear baseline model (M1). GEBVs were estimated strictly within each training fold to prevent data leakage. The resulting input matrix is defined as:


(9) $$X_{\mathrm{in}}=\left[X_{\mathrm{GenLatent}}|X_{\mathrm{EnvPheno}}|X_{\mathrm{EnvLatent}}|X_{G\times E}|{\hat{g}}_{M1}\right]$$

By incorporating ${\hat{g}}_{M1}$, the algorithm receives a strong biological prior representing the global additive structure. This allows the gradient boosting trees to concentrate on modeling residual non-linear patterns and G×E deviations not captured by the linear model.

### Target-oriented learning framework

Unlike previous strategies that minimized prediction error relative to observed phenotypes, this framework modifies the response variable to perform signal transfer. Specifically, a Factor Analytic (FA) mixed model (Smith A et al. 2001) was fitted using *ASReml-R* to isolate the G×E interaction signal. The model is defined as:


(10) $$y=X\beta +Z_{ge}u_{ge}+\epsilon$$

where $u_{ge}$ represents genotype-by-environment interaction effects, assumed as $u_{ge}∼N(0,K⊗\Sigma_{env})$, with K denoting the genomic relationship matrix and ⊗ the Kronecker product. The environmental covariance matrix $\Sigma_{env}$ was modeled using a Factor Analytic structure of order *k*, $\Sigma_{env}=\Lambda \Lambda^{⊤}+\Psi$, where Λ (J×k) is the matrix of factor loadings and Ψ is a diagonal matrix of specific variances.

To account for uncertainty from first-stage BLUE estimation, residual variance was weighted using a diagonal matrix D constructed from the inverse of the estimation error variances, such that $\epsilon ∼N(0,D^{-1}\sigma_{\epsilon }^{2})$. The model order *k* was selected to explain ∼80% of the variance (Smith AB and Cullis 2018). The extracted interaction BLUPs, ${\hat{u}}_{ge}$, were then used as targets for machine learning.

Model M6 employs the same integrated feature space as M4 but is trained to predict the interaction BLUPs rather than total phenotypic values:


(11) $${\hat{u}}_{ge}=f_{\mathrm{XGB}}\left(X_{\mathrm{GenLatent}}|X_{\mathrm{EnvPheno}}|X_{\mathrm{EnvLatent}}|X_{G\times E}\right)$$

While the FA model estimates environment-specific covariance parameters based on observed phenotypes, it cannot directly project interaction effects to unobserved environments. Model M6 overcomes this limitation by learning the latent interaction structure directly from environmental descriptors, enabling extrapolation of complex G×E patterns to new environments based on climatic similarity.

### Ensemble learning via stacked generalization

To leverage the complementarity between global covariance structures captured by statistical models and local non-linear interactions detected by machine learning, a two-level stacked generalization framework was implemented (Wolpert 1992) (Supplementary Fig. S1). This strategy minimizes individual model bias by learning optimal weights across heterogeneous predictive signals (Supplementary Fig. S2).

The first layer of the ensemble (Level 0—Base Learners) consisted of models M1 through M6. The premise of stacking is that distinct algorithms rely on different mathematical approximations. To exploit this synergy, the feature space of the second layer was constructed using fitted values generated by the base models on the training data. To prevent overfitting propagation, gradient boosting models (M3–M6) were trained under strict L1 (Lasso) and L2 (Ridge) regularization, ensuring that fitted values represented stable biological signals rather than noise.

The matrix of predictions generated by Level 0 models is denoted as $P_{L0}$, where each column corresponds to predictions from one base learner. This matrix was used as input for the Level 1 meta-learner, implemented via Ridge regression to address multicollinearity among base predictions. The final stacked prediction is defined as:


(12) $${\hat{y}}_{\mathrm{stack},i}=\beta_{0}+\sum_{m\in S}\beta_{m}{\hat{y}}_{m,i}$$

where ${\hat{y}}_{\mathrm{stack},i}$ denotes the ensemble prediction for individual *i*, ${\hat{y}}_{m,i}$ is the prediction of the *m*th base model, and $\beta_{m}$ represents the Ridge regression coefficients. Regularization was controlled by the hyperparameter $\lambda_{\mathrm{meta}}$, tuned via internal cross-validation within each training fold (Friedman et al. 2010). Rather than constructing a single ensemble including all base models, a systematic combinatorial search was performed evaluating all possible two-way and three-way combinations among M1–M6. The best-performing combinations (top stacks) were selected based on mean predictive ability across environments, enabling identification of the most robust functional synergies for each crop and validation scenario.

### Model evaluation: cross-validation and metrics

To evaluate the robustness and generalization capacity of the proposed models in predicting genotype performance in unobserved environments, we implemented two distinct cross-validation (CV) schemes:

#### Leave-random-environments-out (LREO)

The LREO scheme quantifies the model’s ability to predict genotypes in new locations within the same experimental network. This procedure consisted of 10 independent repetitions. In each iteration, the total set of environments was randomly partitioned: 75% were assigned to the training set and the remaining 25% were used as the validation (test) set. This scenario tests the capacity of the models to interpolate known environmental patterns.

#### Leave-one-year-out (LOYO)

To simulate the challenge of predicting performance in untested years, we applied a LOYO scheme. In this approach, all environments belonging to a specific year were omitted from the training set and used entirely as the validation set. The models were trained using data from all other available years, and the process was repeated for each year in the dataset. This scheme imposes a greater challenge of temporal extrapolation, testing prediction stability against interannual climatic fluctuations and year effects.

#### Evaluation metrics

Predictive ability was defined as the Pearson correlation coefficient (*r*) between the observed phenotypic BLUE vector y and the predicted values $\hat{y}$ within each validated environment:


(13) $$r=\frac{\sum_{i=1}^{n}(y_{i}-\bar{y})({\hat{y}}_{i}-\bar{\hat{y}})}{\sqrt{\sum_{i=1}^{n}(y_{i}-\bar{y}{)}^{2}}\sqrt{\sum_{i=1}^{n}({\hat{y}}_{i}-\bar{\hat{y}}{)}^{2}}}$$

where *n* denotes the number of genotypes evaluated in the validation environment, $y_{i}$ represents the observed BLUE for genotype *i*, and ${\hat{y}}_{i}$ corresponds to its predicted value.

The Root Mean Squared Error (RMSE) was used to quantify the magnitude of prediction error and evaluate scale-dependent accuracy:


(14) $$\mathrm{RMSE}=\sqrt{\frac{1}{n}\sum_{i=1}^{n}(y_{i}-{\hat{y}}_{i}{)}^{2}}$$

where *n* is the number of genotypes in the validated environment.

For final comparisons, performance metrics obtained in each environment were summarized using the arithmetic mean across cross-validation folds and repetitions. This procedure ensures that the reported results reflect the average generalization capacity of each model across the intrinsic volatility of multi-environment trials (MET).

### Statistical characterization of PCA and PaCMAP manifolds

To investigate the biophysical interpretation of latent components, denoted as $Y_{\mathrm{comp}}$ (the E-PC and E-PaCMAP components of $X_{\mathrm{EnvLatent}}$), regression analyses were conducted using environmental features from $X_{\mathrm{EnvPheno}}$ as predictors. This step aimed to identify which climatic variables aggregated by phenological windows most strongly explained variance captured by dimensionality reduction methods.

For each component, the general third-degree polynomial model was defined as:


(15) $$Y_{\mathrm{comp}}=\beta_{0}+\beta_{1}X_{j}+\beta_{2}X_{j}^{2}+\beta_{3}X_{j}^{3}+\epsilon$$

where $X_{j}$ represents the *j*th environmental feature (column) of $X_{\mathrm{EnvPheno}}$. Quadratic and linear models were defined as nested reductions of the full model by constraining higher-order coefficients to zero (i.e. $\beta_{3}=0$ for quadratic; $\beta_{3}=\beta_{2}=0$ for linear models).

Model selection followed statistical parsimony principles using likelihood ratio tests via ANOVA, adopting a significance threshold of α=0.10 to minimize type II error and preserve subtle non-linear biological signals. Higher-order terms were retained only when they significantly improved model fit relative to simpler nested models.

PCA-derived components, which are by construction linear combinations of the environmental features, were evaluated exclusively under linear models. In contrast, PaCMAP components were analyzed under the full polynomial hierarchy, allowing detection of non-linear biological responses and environmental saturation phenomena.

Because latent components, particularly those derived from PaCMAP, represent complex interactions among multiple environmental features, univariate regression was adopted as an exploratory interpretative strategy. Environmental predictors were ranked according to adjusted coefficients of determination ($R_{\mathrm{adj}}^{2}$) and corroborated through visual inspection of trend curves stratified by planting month and location. For maize, locations were partitioned into three equal latitudinal intervals, defined as Southern, Midwestern, and Northern regions, enabling spatial interpretation of environmental gradients.

## Results

### Genetic covariance structure

The modeling of the genetic covariance structure revealed distinct levels of complexity for the G×E interaction in each crop, reflecting the geographic scale and environmental diversity of the experimental networks. For the maize dataset, which covers a continental latitudinal range across the United States and Canada (Fig. 1a), a factor analytic model of order 6 (FA6) was required to explain 79.7% of the total genetic variance, indicating a high-dimensional interaction structure driven by the extensive environmental heterogeneity. In contrast, the rice dataset, restricted to a compact geographic range of approximately 300 km in Uruguay under controlled irrigated conditions (Fig. 1b), exhibited a reduced interaction dimensionality. A factor analytic model of order 2 (FA2) was sufficient to explain 86.4% of the genetic variance. The analysis revealed that while the maize network required multiple latent factors to capture environmental dependencies (FA6), the rice network exhibited a simpler covariance structure where the primary variation was explained by fewer components (FA2).

### Predictive ability across scenarios

In maize and rice, for all validation scenarios (LREO and LOYO), strategies integrating environmental information consistently outperformed the genomic baseline (M1-GBLUP). Relative to M1, the Stacking framework provided gains in predictive ability ranging from approximately 10% in the maize temporal validation (LOYO, r=0.45 vs. 0.41 for M1) to 27.5% in the rice LREO validation (r=0.51 vs. 0.40) (Figs. 3a,b, 4a, 4b). Exact predictive ability and RMSE values for every model, crop and scenario are reported in Table 1.

**Fig. 3.**
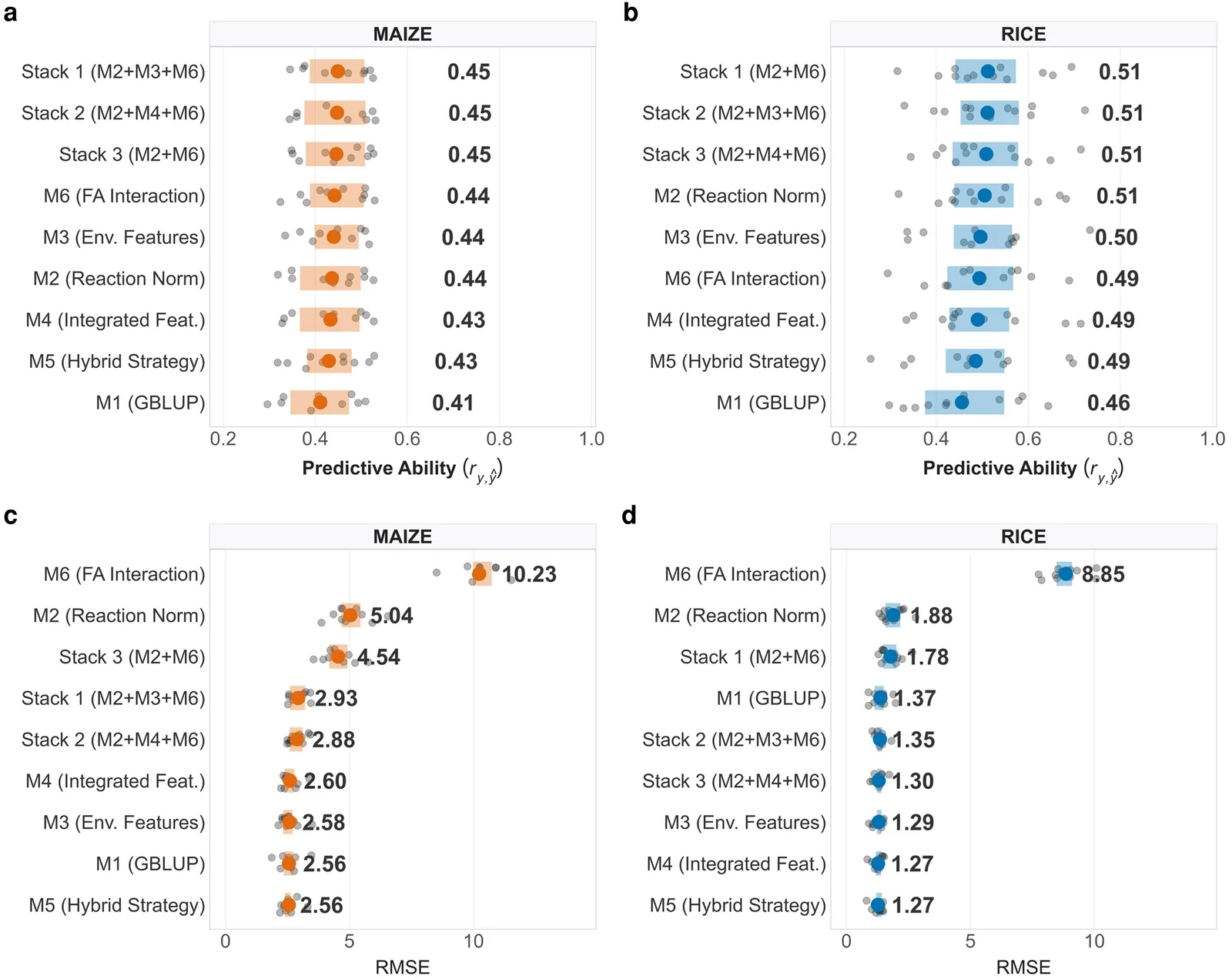
Predictive performance of genomic prediction models assessed via LOYO cross-validation. Panels a) and b) display predictive ability for the maize and rice datasets, respectively. Panels c) and d) present the RMSE for maize and rice datasets, respectively. Boxplots illustrate the distribution of metrics across validation years. The text annotations indicate the mean values across folds.

**Fig. 4.**
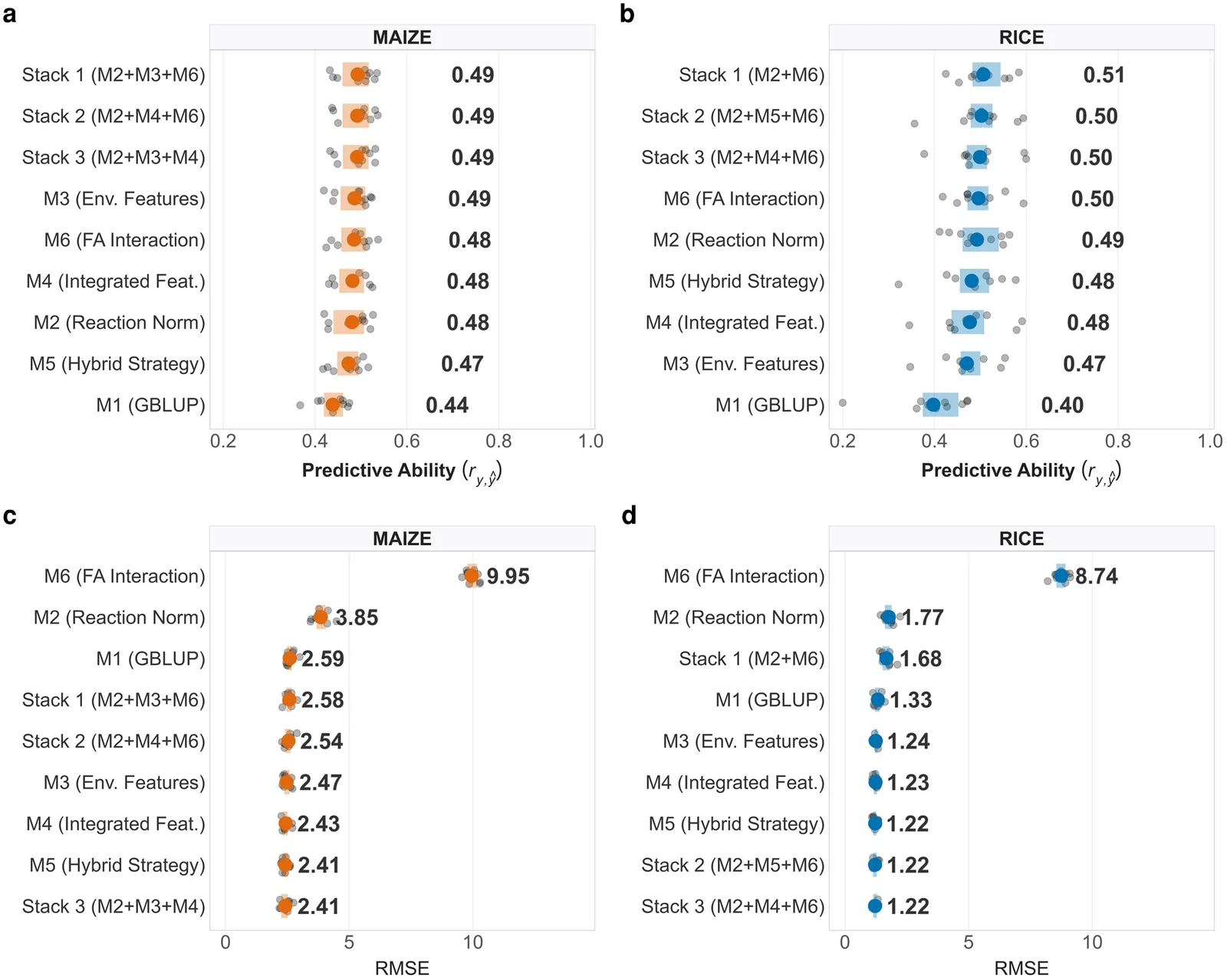
Predictive performance of genomic prediction models assessed via LREO cross-validation. Panels a) and b) display predictive ability for the maize and rice datasets, respectively. Panels c) and d) present the RMSE for maize and rice datasets, respectively. Boxplots illustrate the distribution of metrics across validation folds. The text annotations indicate the mean values across folds.

**Table 1.** Predictive ability (pearson correlation, *r*) and root mean squared error (RMSE) for maize and rice under the two cross-validation schemes (LOYO, leave-one-Year-Out; LREO, Leave-Random-Environments-Out).

		Maize		Rice	
Validation	Model	*r*	RMSE	*r*	RMSE
LOYO	M1 (GBLUP)	0.41 (0.07)	2.56 (0.42)	0.46 (0.11)	1.37 (0.34)
	M2 (Reaction Norm)	0.44 (0.07)	5.04 (0.79)	0.51 (0.11)	1.88 (0.43)
	M3 (Env. Features)	0.44 (0.06)	2.58 (0.39)	0.50 (0.11)	1.29 (0.19)
	M4 (Integrated Feat.)	0.43 (0.07)	2.60 (0.34)	0.49 (0.12)	1.27 (0.19)
	M5 (Hybrid Strategy)	0.43 (0.07)	2.56 (0.33)	0.49 (0.13)	1.27 (0.20)
	M6 (FA Interaction)	0.44 (0.07)	10.23 (0.81)	0.49 (0.11)	8.85 (0.73)
	Ensemble 1 Mz: M2+M3+M6; Rc: M2+M6	0.45 (0.07)	2.93 (0.36)	0.51 (0.11)	1.78 (0.41)
	Ensemble 2 Mz: M2+M4+M6; Rc: M2+M3+M6	0.45 (0.07)	2.88 (0.35)	0.51 (0.11)	1.35 (0.19)
	Ensemble 3 Mz: M2+M6; Rc: M2+M4+M6	0.45 (0.07)	4.54 (0.65)	0.51 (0.11)	1.30 (0.20)
LREO	M1 (GBLUP)	0.44 (0.03)	2.59 (0.17)	0.40 (0.08)	1.33 (0.15)
	M2 (Reaction Norm)	0.48 (0.04)	3.85 (0.32)	0.49 (0.05)	1.77 (0.23)
	M3 (Env. Features)	0.49 (0.04)	2.47 (0.15)	0.47 (0.06)	1.24 (0.08)
	M4 (Integrated Feat.)	0.48 (0.03)	2.43 (0.16)	0.48 (0.07)	1.23 (0.09)
	M5 (Hybrid Strategy)	0.47 (0.03)	2.41 (0.14)	0.48 (0.07)	1.22 (0.09)
	M6 (FA Interaction)	0.48 (0.04)	9.95 (0.25)	0.50 (0.05)	8.74 (0.29)
	Ensemble 1 Mz: M2+M3+M6; Rc: M2+M6	0.49 (0.04)	2.58 (0.17)	0.51 (0.05)	1.68 (0.21)
	Ensemble 2 Mz: M2+M4+M6; Rc: M2+M5+M6	0.49 (0.04)	2.54 (0.17)	0.50 (0.07)	1.22 (0.10)
	Ensemble 3 Mz: M2+M3+M4; Rc: M2+M4+M6	0.49 (0.04)	2.41 (0.18)	0.50 (0.06)	1.22 (0.09)

Each value is the mean across cross-validation folds (LOYO: n=10 yr for maize, n=12 for rice) or across the 10 LREO resampling repetitions (75%/25% train/test split of environments). The value in parentheses is the *between-fold* standard deviation (between-repetition for LREO)—i.e. the fold-to-fold dispersion of the mean metric, not the between-environment standard deviation. Models M1–M6 are defined in the Methods; Ensemble 1–3 are the three best-performing stacked ensembles for each crop, with the base-model composition of each given beneath its label (Mz = maize, Rc = rice; the top ensembles differ between crops because they are ranked per crop). M6 is trained to predict interaction BLUPs rather than the full phenotypic value, which inflates its RMSE.

Among the individual strategies, the Enviromic Reaction Norm (M2) and the interaction-learning XGBoost (M6) consistently ranked as the top predictors. The feature-based machine learning strategies (M3, M4, and M5) exhibited comparable performance levels, forming a distinct cluster with similar predictive accuracies across all scenarios. The engineering of explicit interaction terms (M4) and the incorporation of GEBVs (M5) yielded results equivalent to the base phenological feature set (M3). Finally, the ensemble approach refined these predictions, with small gains over the reaction norm model (M2) ranging from 0% (a tie in rice LOYO) to 4% (0.51 vs. 0.49 in rice LREO), depending on the crop and validation scheme.

### Predictive ability vs. RMSE

The analysis of the RMSE revealed a divergence between predictive ability and scale accuracy (RMSE). While the model M6 and the M2 achieved high correlations, they consistently presented the highest RMSE values across datasets, an expected outcome given that M6 is trained to predict interaction effects (BLUPs) rather than the full phenotypic scale. Notably, despite these high individual error rates, the integration of M2 and M6 within the stacking framework resulted in a composite prediction with significantly reduced error compared to the isolated models.

The magnitude of RMSE reduction provided by the ensembles depended on the selection criterion. When selecting the top ensembles based on predictive ability, RMSE reductions relative to the M2 baseline ranged from approximately 5% in rice (LOYO and LREO: 1.78 vs. 1.88 and 1.68 vs. 1.77), up to 42% in maize (LOYO: 2.93 vs. 5.04). However, optimizing the ensemble selection for RMSE minimization had a negligible impact on predictive ability while generating more consistent gains across all datasets. In this context, RMSE reductions ranged from 30–31% in rice (1.30 vs. 1.88 in LOYO; 1.22 vs. 1.77 in LREO) and 37–43% in maize (2.88 vs. 5.04 in LOYO; 2.41 vs. 3.85 in LREO) compared to the M2 baseline.

### Feature importance

The analysis of feature importance, based on the mean gain metric of the XGBoost algorithm (M6), quantified the relative contribution of genomic, environmental, and interaction predictors (Fig. 5). The first genomic PaCMAP component (G-PaCMAP 1) consistently ranked as the top predictor across both crops and validation schemes. However, the linear global structure captured by PCA components remained highly relevant, frequently occupying the subsequent top positions alongside the non-linear manifold features.

**Fig. 5.**
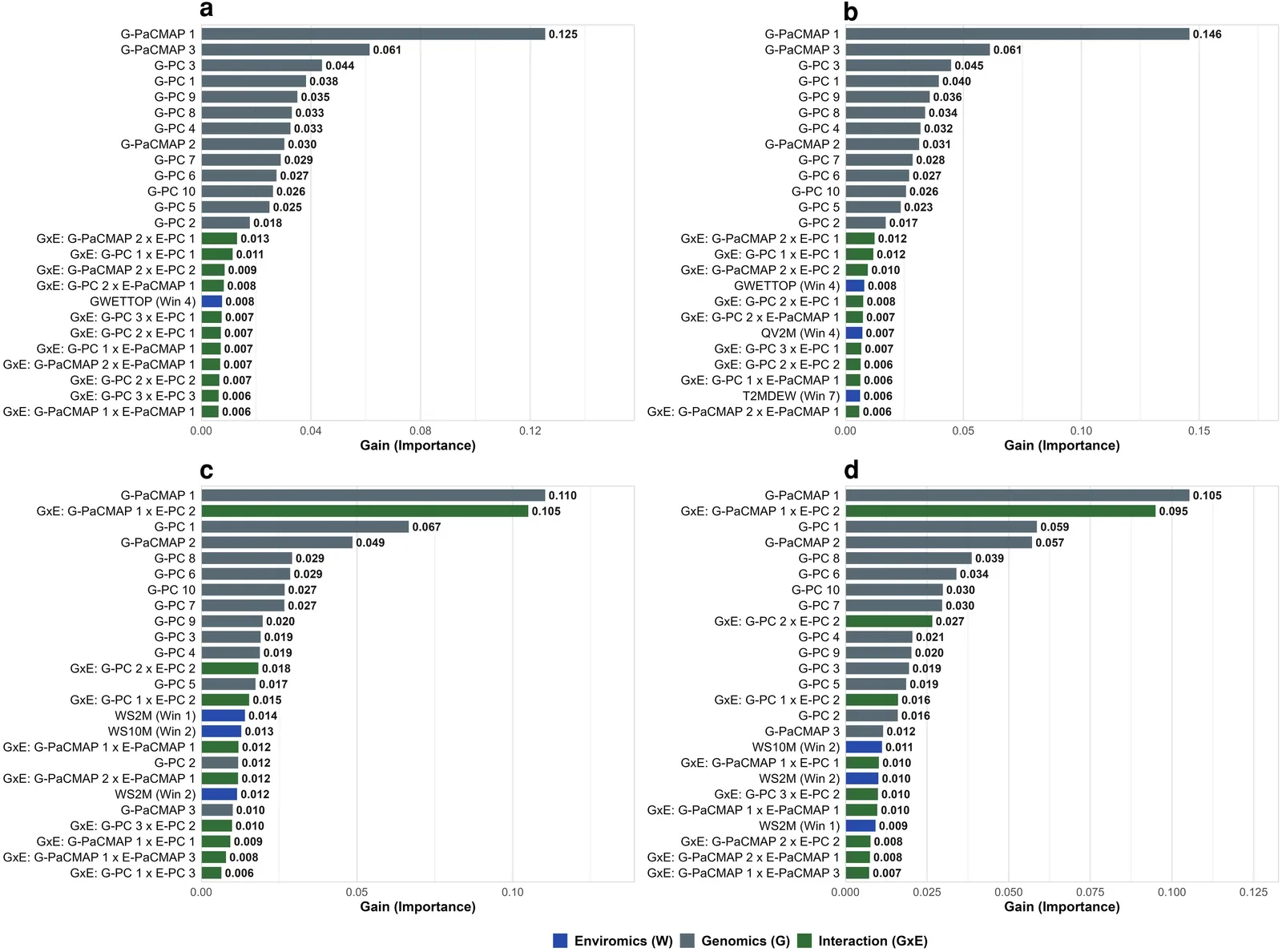
Relative importance of the top 25 predictor features used by the M6 model. Upper panels display results for the Maize crop under a) LOYO and b) LREO validation schemes. Lower panels display results for the rice crop under c) LOYO and d) LREO. Feature importance was calculated based on the mean gain metric of XGBoost across validation folds, which measures the relative contribution of each feature to the reduction of the loss function. Colored bars indicate the feature category: Genomics (Gray; e.g. G-PC, G-PaCMAP), Enviromics (Blue; e.g. E-PC, E-PaCMAP, Weather Features), and G×E Interaction (Green; e.g. G×E: G-PC × E-PC).

Regarding the environmental and interaction signals, the explicit interaction terms (G×E) generated by the element-wise multiplication of latent genomic and environmental factors exhibited substantial importance, often appearing within the top 15 predictors. In contrast, pure environmental features generally showed lower relative gain compared to the composite interaction terms and the latent genomic descriptors.

### Latent component interpretation

Regression analysis enabled the interpretation of the biophysical meaning of the most important environmental components for the M6 model (Fig. 5; Table 2). In maize, the first principal component (E-PC 1) showed a strong linear relationship ($R_{\mathrm{adj}}^{2}\approx 0.86$) with temperature and solar radiation features (Supplementary Fig. S3). The plots revealed a clear geographic division: Northern environments clustered at low values, followed by the Midwest, and finally the South at higher values. Additionally, this latitudinal separation coincided with a clear temporal pattern, where southern environments were predominantly associated with earlier planting dates compared to northern trials.

**Table 2.** Environmental features explaining the latent components (E-PC 1–2 and E-PaCMAP 1–2) for each crop.

Crop	Component	Environmental drivers (window)	Model	$R_{adj}^{2}$
Maize	E-PC 1	ALLSKY_SFC_LW_DWN (W7), T10M (W7), T2M (W7), T2M_MIN (W7)	Linear	0.86–0.87
Maize	E-PC 2	VPD (W2, W5), RH2M (W5, W6)	Linear	0.71–0.78
Maize	E-PaCMAP 1	SPV (W6), T10M (W5, W6), T2M (W6)	Quadratic / Cubic	0.64–0.65
Maize	E-PaCMAP 2	SPV (W5), T10M (W5), T2M (W5), T2M_MIN (W5)	Cubic / Quadratic	0.30–0.32
Rice	E-PC 1	T10M (W2), T2M (W2), SPV (W2), T2M_MIN (W2)	Linear	0.74–0.81
Rice	E-PC 2	N (W1), CLRSKY_SFC_SW_DWN (W1), RTA (W1, W3)	Linear	0.69–0.84
Rice	E-PaCMAP 1	T10M (W3), WS10M (W1), WS2M (W3), T2M (W3)	Linear / Quadratic	0.41–0.44
Rice	E-PaCMAP 2	T10M (W3), T2M (W3), SPV (W3), WS10M (W1)	Linear / Quadratic	0.26–0.29

For each component, the leading explanatory features (with their phenological window), the fitted model form, and the range of adjusted $R^{2}$ across those features are reported. Full regressions are shown in Supplementary Figs. S3–S6.

Variations in water deficit were captured by the second component (E-PC 2), showing a high linear correlation ($R_{\mathrm{adj}}^{2}\approx 0.78$) with Vapor Pressure Deficit (VPD) and Relative Humidity (RH2M). Unlike E-PC 1, this component did not separate geographic regions, presenting substantial overlap among environments from the three zones along the axis.

The first PaCMAP component (E-PaCMAP 1) revealed non-linear relationships, fitting better to quadratic and cubic models ($R_{\mathrm{adj}}^{2}\approx 0.65$) with the Slope of Vapor Pressure (SPV) and mean temperature (Supplementary Fig. S4). The data revealed a non-linear response with diminishing returns, highlighting different levels of atmospheric demand. The Northern region exhibited significant variation along the initial (ascending) trajectory of the curve, indicating that PaCMAP successfully differentiated sub-environments within this latitude, ranging from low-demand locations (cold and wet) to locations experiencing moderate stress. Conversely, the Southern region was concentrated in the stable portion (plateau) of the curve, characterizing a constant regime of high water demand (evapotranspiration).

In the rice dataset, variable importance analysis revealed a clear hierarchy: the temporal dimension (sowing date) predominated over the spatial dimension (location), albeit with significant microclimatic adjustment.

Although E-PC 1 captures the largest portion of environmental variance, the variable importance analysis revealed that E-PC 2 was a primary driver of predictive ability, exhibiting a strong linear relationship ($R_{\mathrm{adj}}^{2}\approx 0.69\text{--}0.84$) with photoperiod and solar radiation (Supplementary Fig. S5). Visual inspection of the plots showed a distinct temporal gradient: early sowing environments (September) clustered at one extreme and progressed linearly toward late-season environments (December), confirming this axis as a proxy for phenological stages driven by photoperiodic variation across sowing dates.

At the second level of importance, E-PC 1 captured variations in temperature and water demand, correlating linearly with mean temperature and the slope of the vapor pressure curve ($R^{2}\approx 0.74$–0.81). Unlike the previous component, E-PC 1 resulted in a distinct geographic separation, isolating the Paso Farías location, which presented thermal regimes distinct from the other sites.

Finally, the first PaCMAP component (E-PaCMAP 1) reinforced the geographic identification of Paso Farías (Supplementary Fig. S6). This component was explained by linear and quadratic relationships with wind speed and temperature ($R_{\mathrm{adj}}^{2}\approx 0.43$–0.44). Data distribution indicates that PaCMAP isolated Paso Farías based on its specific microclimate, capturing a unique interaction between wind and temperature that differentiates this location from others regardless of sowing date.

### Experimental quality

The relationship between predictive ability and experimental quality metrics ($H_{\mathrm{Cullis}}^{2}$ and $CV_{e}$) varied by crop (Fig. 6). In maize, which featured a broad spectrum of trial qualities, significant correlations were observed: predictive ability increased with heritability (r=0.52, p<0.001) and decreased with residual error (r=−0.25, p<0.001). In contrast, the rice dataset exhibited distinct patterns due to range restriction. For heritability, no significant correlation was detected, as trials were predominantly of high quality, displaying clustered distributions with scarcity of environments presenting $H_{\mathrm{Cullis}}^{2}<0.4$. The same pattern held for the residual coefficient of variation ($CV_{e}$), for which no significant association was detected (p=0.89), again reflecting the limited variability among trials: the frequency distribution indicated a scarcity of trials with $CV_{e}$ exceeding 15%.

**Fig. 6.**
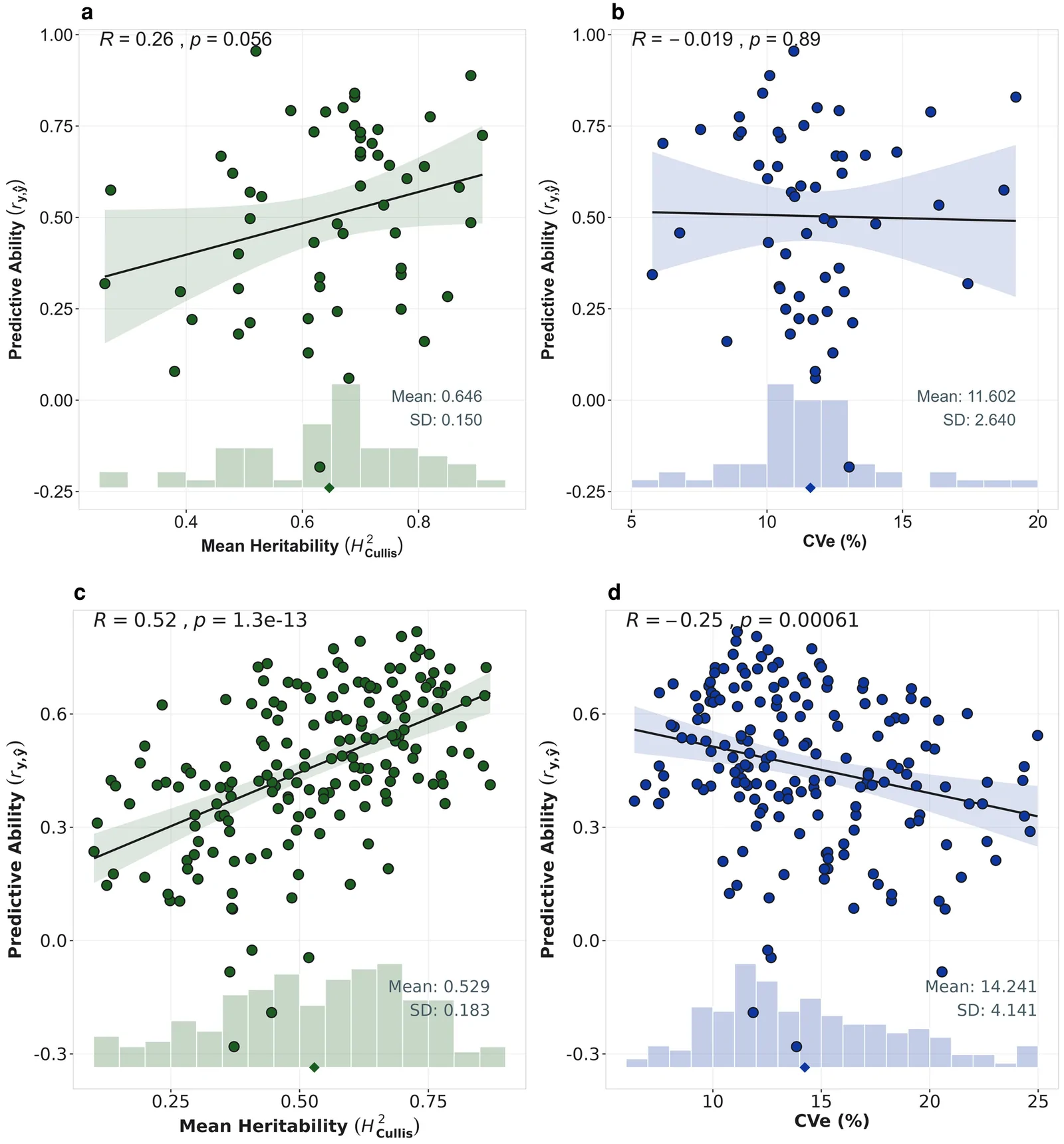
Influence of experimental quality metrics on predictive ability in rice and maize. The left column presents scatter plots of predictive ability from LOYO versus heritability ($H_{\mathrm{Cullis}}^{2}$) for rice and maize (A, C). The right column presents scatter plots of predictive ability from LOYO versus residual coefficient of variation ($CV_{e}$) for rice and maize, respectively. Each panel includes an integrated background histogram displaying the frequency distribution of the corresponding x-axis metric ($H_{\mathrm{Cullis}}^{2}$ or $CV_{e}$). Solid lines represent the linear regression fit with 95% confidence intervals. Statistical annotations include the Pearson correlation coefficient (*r*), *p*-value (*p*), and the mean and standard deviation (SD) of the quality metrics.

## Discussion

### G×E dimensionality

The divergence in the optimal covariance structures between maize and rice suggests that the dimensionality of G×E interaction is intrinsically linked to the nature of the environmental drivers predominant in each experimental network. In the maize dataset, the requirement for a higher-order model (FA6) is attributable to the continental scale of the trials, where massive photoperiodic and thermal variations create different interaction patterns across the MET. This high dimensionality is consistent with observations in tropical environments. Chaves et al. (2023), evaluating maize hybrids across a geographically diverse network of trials in Brazil that included significant latitudinal variation, found that an FA4 model was required to capture the interaction caused by contrasting sowing seasons, using a set of 48 environments. This finding justifies the need for a higher-order model (FA6) in our study, particularly considering the substantial increase to 180 environments.

In contrast, the FA2 model was sufficient for rice in Uruguay, suggesting more uniform growing conditions driven by controlled irrigation that prevents water deficit. However, the factor analytic structure was still necessary, explaining a high proportion of genetic variance and demonstrating that even in spatially compact and managed environments, latent factors continue to generate interaction. In this context, the primary source of environmental variation shifts from spatial (geography) to temporal (years) and management-driven factors, such as sowing dates.

### Predictive gains and stacking

The results confirm that integrating enviromics into genomic prediction provides consistent gains over the GBLUP baseline (M1). Among the strategies that incorporate climatic data (M2 to M6 and the stacking ensembles), predictive ability was broadly comparable: the enviromic reaction norm (M2) and the interaction-learning model (M6) were among the top performers, and the stacking framework added only small gains over the best individual models. The performance of M6 is consistent with Araújo et al. (2024), showing that factor analytic inference with enviromic data can capture environmental dependencies.

Ensemble strategies that combine multiple base models through a meta-learner, such as EXGEP, have recently been applied to genomic prediction (Nascimento et al. 2024; Yu et al. 2025; Celeri et al. 2026). In our data, the Ridge meta-learner of the best ensembles assigned substantial weight to M2 and M6 (Supplementary Fig. S2); however, the combination raised predictive ability only marginally over the best individual model. Some individual models particularly M3 through M5 achieved an even lower RMSE, albeit with marginally lower predictive ability. The ensemble maintained the high predictive ability of models M2 and M6 while reducing RMSE compared to them, positioning itself among the best models on both metrics and decreasing reliance on the selection of a single best performing model.

Finally, the ensemble approach was crucial to resolve the difference between predictive ability and scale accuracy (RMSE). The interaction-learning model (M6) consistently presented the highest RMSE across datasets. This result stems from the target variable: M6 predicts interaction BLUPs rather than the full phenotypic value. For practical application beyond ranking, phenotype reconstruction would be required by adding the predicted interaction to the environmental mean, a strategy similar to that adopted by the Corteva Latin America Corn (CLAC) team in the Genomes to Fields competition (Washburn et al. 2025). However, the stacking framework automatically corrected this scale issue by integrating the stability of feature-based models (M3, M4, and M5), allowing the final prediction to retain high predictive ability while minimizing error. In applied breeding, the single-model reaction norm (M2) may be simpler to implement than stacking models, reaching comparable and robust predictive ability, but the stacking framework may go further, mainly by reducing the RMSE.

### Feature engineering and manifolds

The XGBoost models (M3 to M6) performed comparably. Although the differences in predictive ability among them were narrow, the feature-importance analysis (Fig. 5) shows which features the model used most. Beyond phenological windowing, the inclusion of PaCMAP alongside PCA components provided an additional set of latent predictors. Notably, the first genomic PaCMAP component (G-PaCMAP 1) consistently ranked as the top predictor across crops and validation schemes, above the traditional principal components.

Furthermore, the high relative importance of the explicit interaction terms (green bars in Fig. 5) indicates that the model used the interaction features generated by the element-wise multiplication strategy. These findings align with recent studies (Godoy Filho et al. 2026b), indicating that non-linear dimensionality reduction serves as a complementary information layer to PCA. The utility of PaCMAP in capturing complex biological topologies has been further corroborated in diverse fields, ranging from multi-omics integration for cancer prognosis (Qattous et al. 2024) to the unsupervised identification of viral lineages in large genomic datasets (Cahuantzi et al. 2024). Taken together, these results suggest that the local topological structure of genomic and environmental data, preserved by manifold learning, can be as effective as or superior to the global variance captured by traditional linear methods in predicting phenotypic plasticity.

### Environmental drivers

The latent factors most important to the M6 model help interpret the environmental features underlying its predictions. In maize, E-PC 1 effectively acted as a proxy for regional adaptation, capturing the latitudinal gradient and differentiating hybrids adapted to distinct thermal and photoperiodic zones. Crucially, this spatial separation was also linked to management decisions, as the thermal gradient mirrored the planting schedule: southern environments were characterized by earlier sowing dates compared to the late-planting northern trials (Supplementary Fig. S3b).

Concurrently, E-PC 2 captured atmospheric water stress. The absence of regional stratification in this component suggests that it allows the model to differentiate genotypic sensitivity to aridity independently of latitude, identifying dry or wet environments across any agroecological zone. However, the curvilinear relationship of E-PaCMAP 1 with the Slope of Vapor Pressure (SPV) indicates that the response to water stress possesses a critical non-linear component. Unlike E-PC 2, PaCMAP presented a clearer latitudinal separation, but with a fundamental distinction: the segregation of northern environments into two stress strata (low vs. medium demand). This result suggests that the linear signal of E-PC 2, while useful, was insufficient to fully model genotype reaction norms. The inclusion of the non-linear topology of PaCMAP was necessary to capture intra-regional physiological nuances that refine the prediction of phenotypic plasticity.

The hierarchy of latent components in rice indicates that the model prioritized temporal characterization (sowing date) over spatial characterization (location) to maximize predictive ability. The predominance of E-PC 2 as the main environmental driver interacting with genomic components validates the strategy of the INIA rice breeding program, which employs staggered sowing dates over time. By linearly capturing photoperiod and available radiation, this result suggests that sowing date is a crucial source of G×E interaction that can be exploited to increase yield (Rebollo et al. 2023).

Geographic characterization was achieved in a complementary manner by component E-PC 1, which differentiated the Paso Farías location through linear thermal variations. However, the fact that E-PaCMAP 1 was also utilized as an important feature in the model suggests that linear norms were insufficient to describe the singularity of this environment. The PaCMAP component isolated Paso Farías based on its specific microclimate, likely capturing a characteristic wind-temperature pattern that distinguishes this site regardless of planting season.

Ultimately, the integration of latent features derived from PCA and PaCMAP enables an approximation of a complex adaptive topography, consistent with the theoretical framework of fitness and crop performance landscapes, in which adaptive peaks emerge from the interaction between genetic architecture and environmental (Wright 1932; Messina et al. 2011).

### Experimental quality as a limit

The relationship between predictive ability and experimental quality metrics ($H_{\mathrm{Cullis}}^{2}$ and $CV_{e}$) reinforces that genomic prediction is bounded by the reliability of phenotypic data. In maize, the significant correlations observed confirm that environments with higher heritability and lower residual error provide clearer biological signals. In contrast, the lack of statistical significance for heritability in rice is attributable to range restriction, as the trials were predominantly of high quality.

This observation aligns with empirical findings by Combs and Bernardo (2013), who demonstrated that traits with higher heritability consistently yield higher prediction accuracies when population size and marker density are held constant. Therefore, the lack of statistical significance in our rice dataset reflects a clustered distribution of high-quality data rather than the absence of the underlying biological relationship. Consequently, these results underscore a fundamental imperative for breeding programs: maximizing predictive potential requires not only advanced modeling but also rigorous experimental quality control to ensure heritability, alongside strategies to expand genetic diversity that effectively increase exploitable genetic variance.

### Future directions

Future research should focus on expanding the ensemble architecture by incorporating a broader spectrum of algorithms as Level 0 base learners, including deep learning architectures, diverse machine learning models, and distinct Bayesian approaches. Increasing the diversity of mathematical approximations at the base level may provide a more informative input space for the Level 1 meta-model. Furthermore, the promising results obtained with PaCMAP suggest that non-linear dimensionality reduction methodologies warrant deeper investigation within genomic and enviromic prediction. These tools should be applied to biologically dissect the environmental drivers of G×E interaction, facilitating the identification of interaction patterns. Finally, studies on hyperparameter optimization for manifold learning techniques are necessary to maximize their interpretability and stability across different crop species and environmental scenarios.

## Conclusions

The results of this study indicate that integrating enviromics into genomic prediction models provides consistent gains in predictive ability compared to the GBLUP baseline, in both new location (LREO) and new year (LOYO) scenarios. The joint utilization of factor analytic inference, enviromics, and machine learning allowed for the capture of distinct components of G×E interaction, suggesting that modeling latent genetic correlations via FA provides a robust biological signal that can be efficiently processed by algorithms to predict performance in unobserved environments. Stacked Generalization emerged as a robust framework for multi-environment prediction, the employment of a meta-model to combine diverse statistical and computational bases contributed to the stabilization of predictions, effectively mitigating individual model selection bias. Crucially, this approach delivered modest gains in predictive ability accompanied by a reduction in RMSE.

Regarding high-dimensional data processing, the high importance of PaCMAP features highlights the value of manifold learning techniques as useful complementary tools to traditional PCA. These results suggest that PaCMAP, alongside other recently developed dimensionality reduction methodologies, demonstrates significant potential to capture complex data structures that linear methods might miss, supporting their broader application in plant breeding. In conclusion, while new modeling architectures and feature engineering approaches expand predictive capacity, performance remains intrinsically bounded by experimental data quality. No model proved capable of overcoming low heritability or excessive experimental noise. Therefore, the advancement of genomic prediction depends on the synchronous evolution of statistical-computational models and the rigorous conduct of phenotypic trials and data collection.

## Supplementary Material

jkag257_Supplementary_Data

## Data Availability

All data required to reproduce the results of this study, comprising the phenotypic, genomic, and enviromic data for both the maize and rice datasets, are available in the public repository at https://github.com/labdgmesalq/Enviromic_genomic. All datasets are provided as R data (.Rdata) files, and the repository also includes example codes showing how to reproduce the models and provides the DOI https://doi.org/10.5281/zenodo.19870083 (Godoy Filho et al., 2026a), under which the archived data are permanently deposited. Supplemental material available at G3 online.
