# Supplementary material for: Stacked enviromic–genomic models improve prediction of genotype performance in new environments
Source: G3 (Bethesda). 2026 Sep 18;16(11):jkag257. doi: 10.1093/g3journal/jkag257 (PMC13732439; doi:10.1093/g3journal/jkag257)
Supplement: Supplementary file 1 [file jkag257_supplementary_data.pdf]

SUPPLEMENTARY

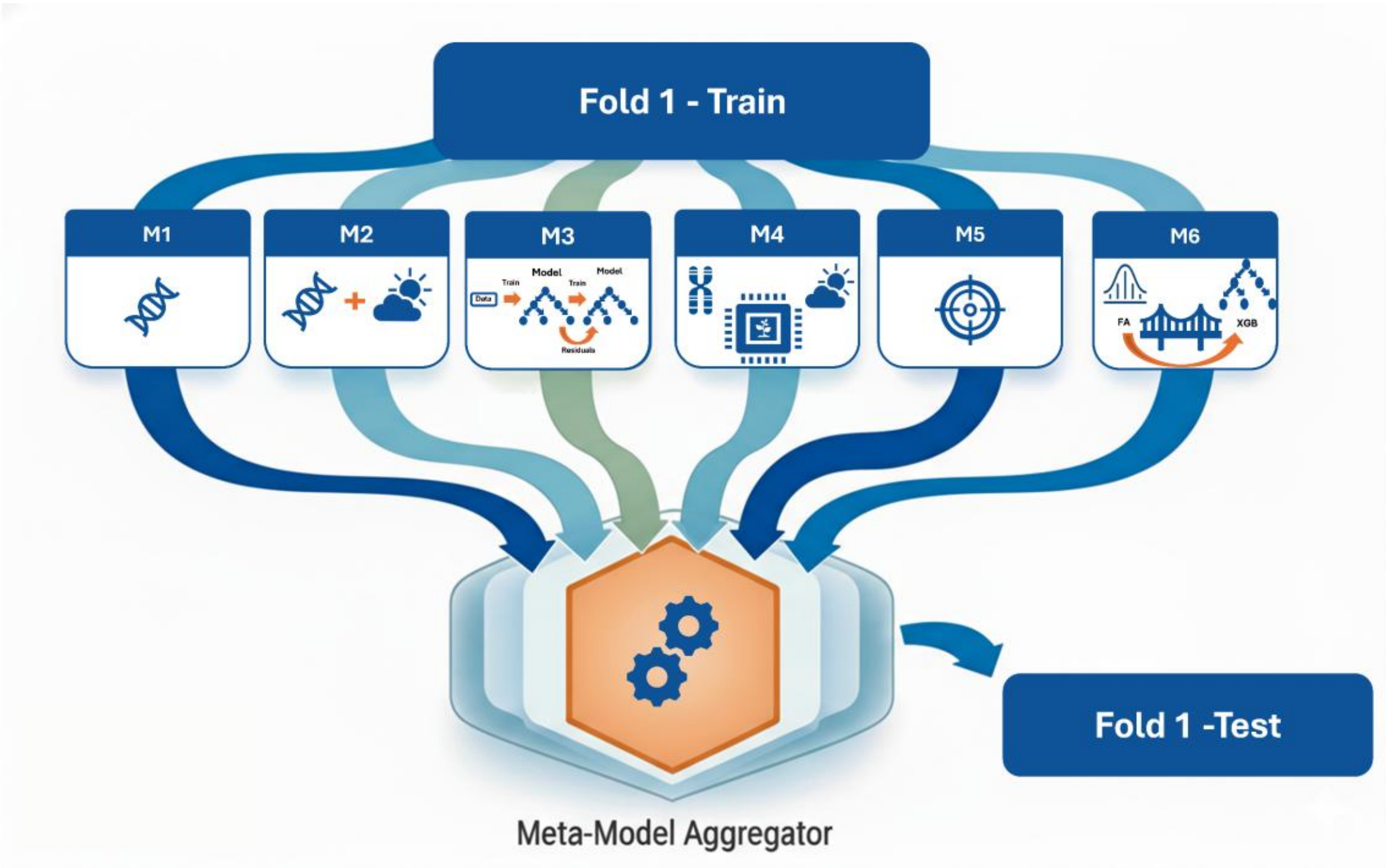

Supplementary Figure S1. Schematic representation of the stacking ensemble learning framework. The workflow illustrates the two-level hierarchical modeling approach. Level 0 explores a pool of six base models (M1 to M6) representing distinct predictive strategies. To optimize predictive performance, a combinatorial search evaluates 2 way and 3-way arrangements of these models. In each validation fold (e.g., Fold 1 - Train), predictions from the base model combinations serve as input features for the Level 1 Meta-Model Aggregator. This aggregator, implemented via Ridge Regression, optimizes the regression coefficients assigned to each base strategy to minimize error and maximize predictive ability in the independent validation set (Fold 1 - Test).

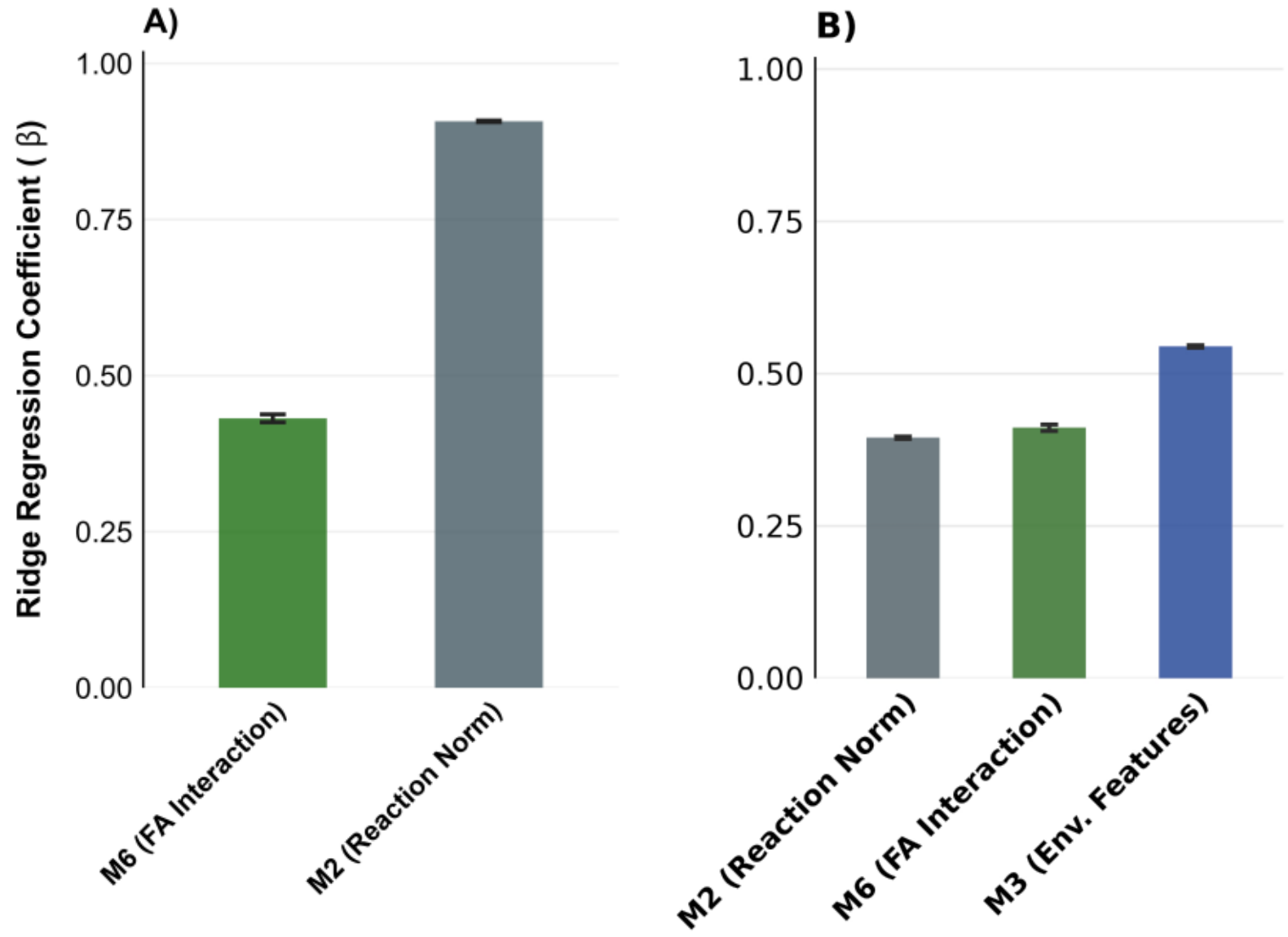

Supplementary Figure S2. Importance of base models in the top stacking ensemble. (A) Mean ridge regression coefficients (β) for the rice dataset across LOYO validation folds. Bars represent the weights assigned by the meta-learner to each base strategy, where higher values indicate a greater influence on the final ensemble prediction. The non-zero weights across different architectures highlight the synergy between linear mixed models and machine learning. Error bars represent the standard error of the mean. Panel (B) follows the same layout for the maize dataset.

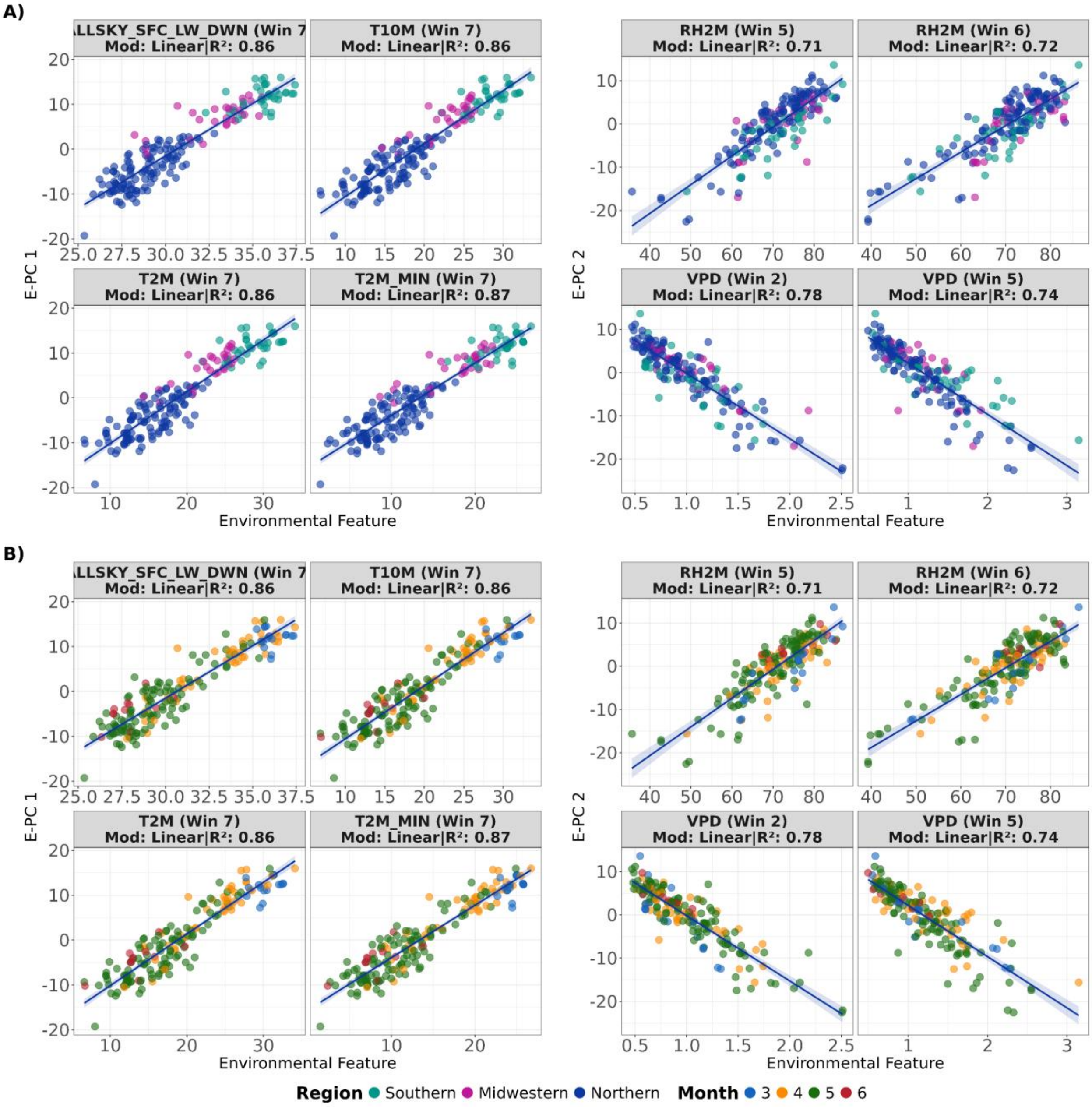

Supplementary Figure S3. Biophysical drivers of the environmental Principal Component Analysis (PCA) in Maize. Scatter plots illustrating the linear relationships between the first two latent environmental principal components (E-PC 1 and E-PC 2) and the top explanatory climatic features. (**A**) Data points are colored by geographic region (Northern, Midwestern, and Southern), highlighting the strong latitudinal gradient captured by E-PC 1 (Temperature/Radiation). (**B**) Data points are colored by planting month. The solid blue line represents the linear regression fit, and the shaded area indicates the 95% confidence interval. Feature acronyms, phenological windows, and adjusted R^2 values are provided in the facet headers.

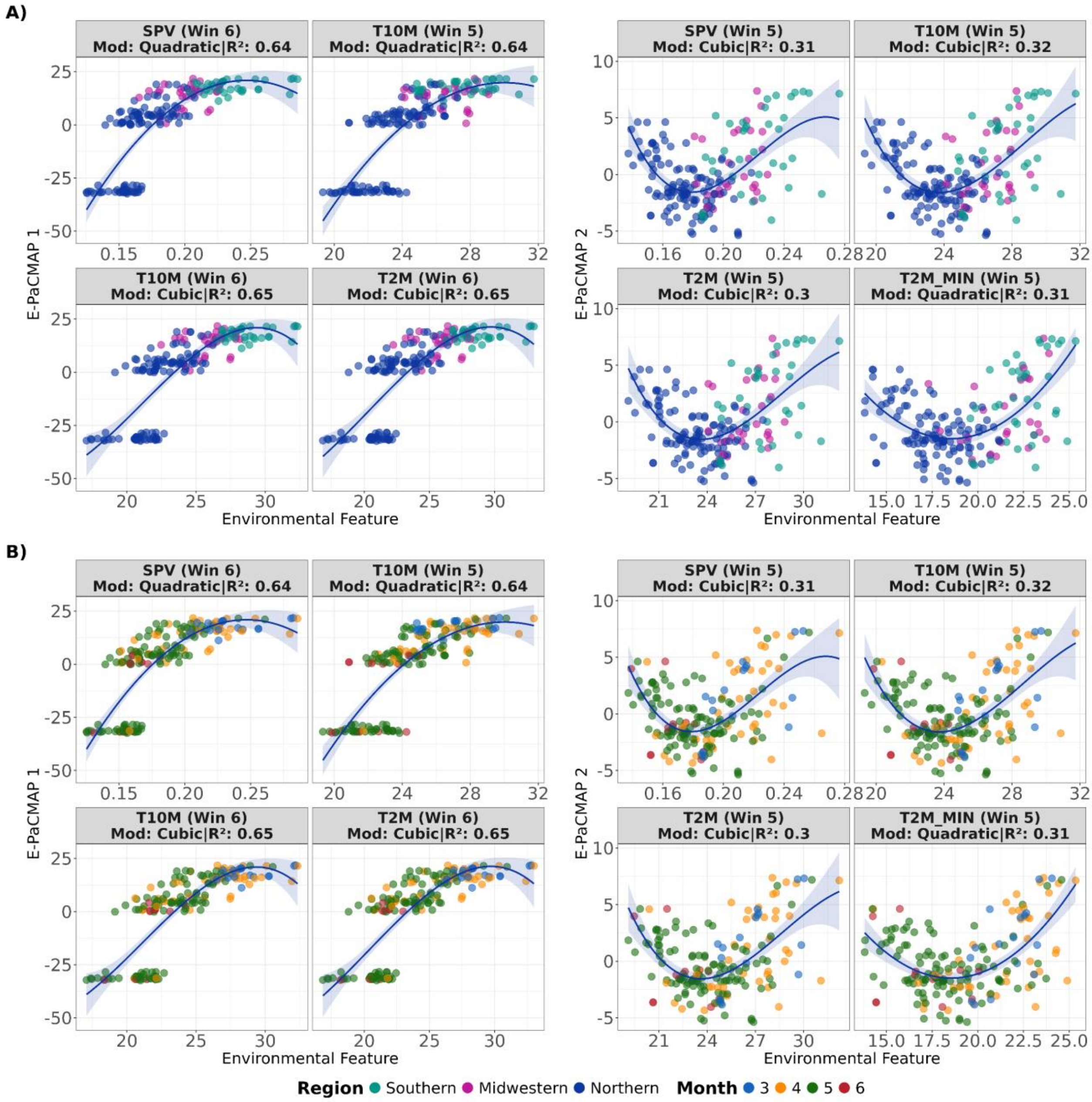

Supplementary Figure S4. Biophysical drivers of the environmental PaCMAP manifold in **Maize.** Scatter plots illustrating the non-linear relationships between the first two PaCMAP components (E-PaCMAP 1 and E-PaCMAP 2) and key environmental drivers. Unlike PCA, relationships were modeled using polynomial regression (Quadratic or Cubic) to capture biological saturation points. (**A**) Data points are colored by geographic region, revealing distinct stress response curves, particularly in the Northern region within E-PaCMAP 1. (**B**) Data points are colored by planting month. The solid blue line represents the polynomial regression fit, and the shaded area indicates the 95% confidence interval.

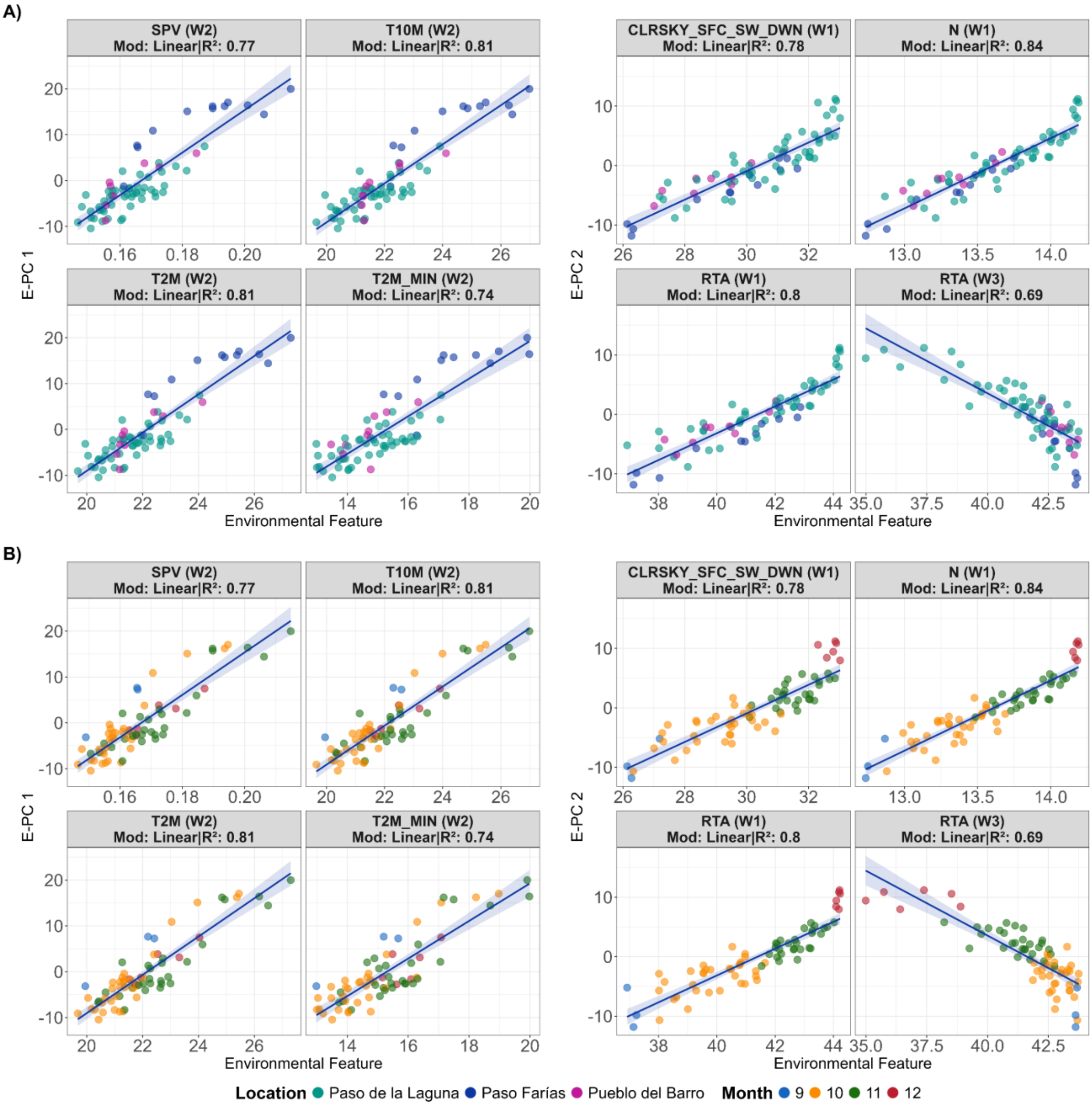

Supplementary Figure S5. Biophysical drivers of the environmental Principal Component Analysis (PCA) in Rice. Scatter plots illustrating the linear relationships between the first two latent environmental principal components (E-PC 1 and E-PC 2) and the top explanatory climatic features. (**A**) Data points are colored by trial location (Paso de la Laguna, Paso Farías, and Pueblo del Barro), showing the isolation of Paso Farías by E-PC 1 based on thermal regime. (**B**) Data points are colored by sowing month, highlighting the near-perfect temporal gradient captured by E-PC 2 (Photoperiod/Radiation), which acts as a phenological proxy. The solid blue line represents the linear regression fit with a 95% confidence interval.

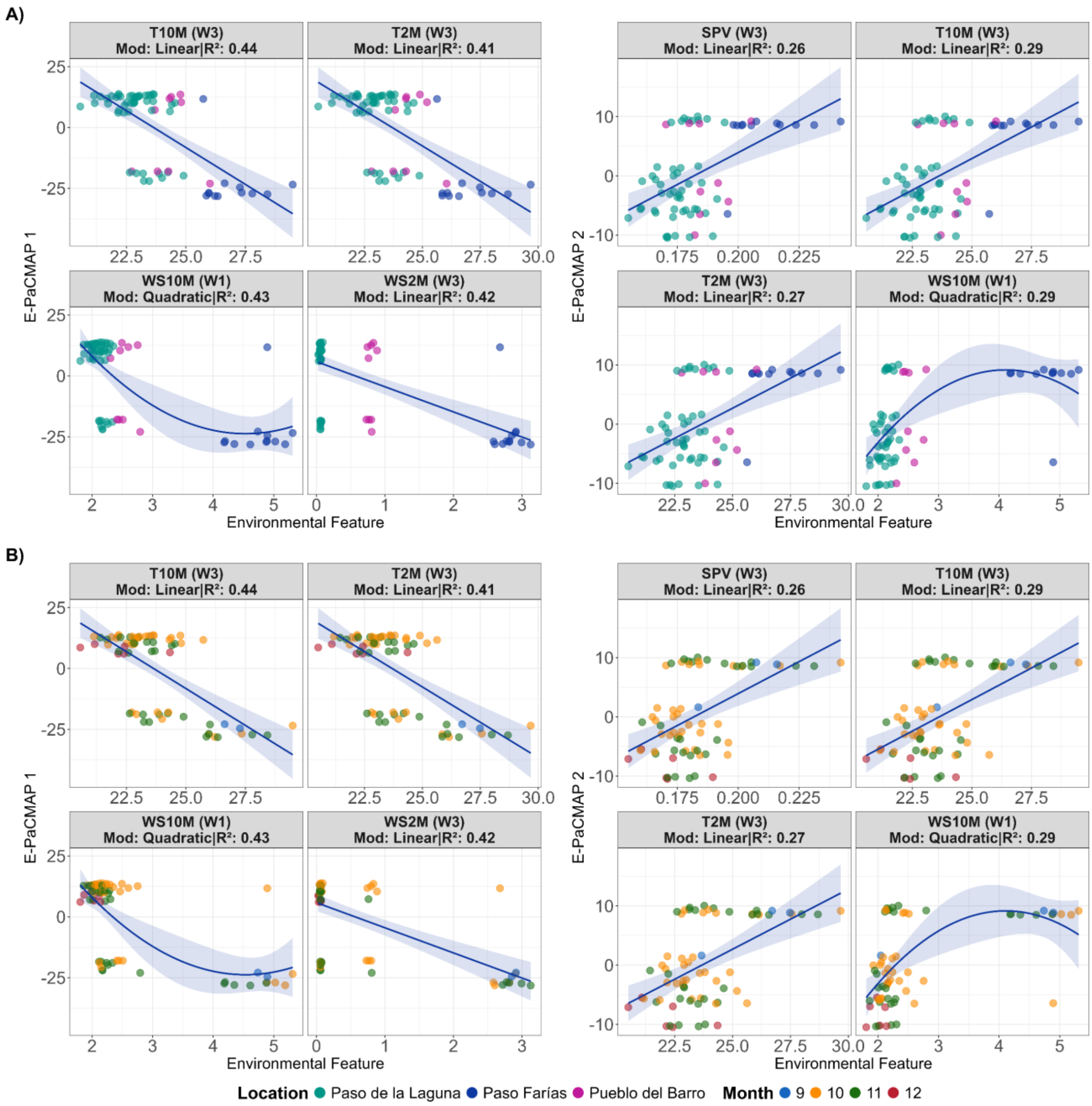

Supplementary Figure S6. Biophysical drivers of the environmental PaCMAP manifold in Rice. Scatter plots illustrating the non-linear relationships between the first two PaCMAP components (E-PaCMAP 1 and E-PaCMAP 2) and key environmental drivers. (**A**) Data points are colored by trial location, emphasizing the microclimatic specificity of Paso Farías (Wind Speed/Temperature interactions) captured by E-PaCMAP 1. (**B**) Data points are colored by sowing month. The solid blue line represents the polynomial regression fit (Quadratic or Cubic), and the shaded area indicates the 95% confidence interval

Supplementary Table S1: Comprehensive list of the 33 environmental features utilized to compose the phenological feature matrix ($\boldsymbol{X}_{EnvPheno}$), in which values are aggregated by specific phenological windows. The Processing/Source column distinguishes between raw daily meteorological parameters obtained from the NASA POWER platform and derived agrometeorological indices such as Growing Degree Days (GDD) and Vapor Pressure Deficit (VPD) also computed on a daily basis via the *EnvRtype* package (Costa-Neto et al., 2021) prior to their temporal aggregation into the respective developmental windows

Category	Acronym	Description	Processing/Source
Temperature	T2M	Temperature at 2 Meters	NASA
	T2M_MAX	Maximum Temperature at 2 Meters	NASA
	T2M_MIN	Minimum Temperature at 2 Meters	NASA
	T10M	Temperature at 10 Meters	NASA
	T2MDEW	Dew/Frost Point at 2 Meters	NASA
	T2M_RANGE	Daily Temperature Range	EnvRtype
	GDD	Growing Degree Days	EnvRtype
Precipitation & Hydrology	PRECTOT	Corrected Total Precipitation	NASA
	EVLAND	Evaporation Land	NASA
	EVPTRNS	Evapotranspiration Energy Flux	NASA
	ETP	Potential Evapotranspiration	EnvRtype
	PETP	Deficit by Precipitation (P - ETP)	EnvRtype
Radiation & Light	ALLSKY_SFC_SW_DWN	All Sky Surface Shortwave Downward Irradiance	NASA
	CLRSKY_SFC_SW_DWN	Clear Sky Surface Shortwave Downward Irradiance	NASA
	ALLSKY_SFC_LW_DWN	All Sky Surface Longwave Downward Irradiance	NASA
	ALLSKY_SFC_PAR_TOT	All Sky Surface Total PAR	NASA
	RTA	Extraterrestrial Radiation	EnvRtype
	n	Actual duration of sunshine	EnvRtype
	N	Daylight hours (Photoperiod)	EnvRtype
Humidity & Air	RH2M	Relative Humidity at 2 Meters	NASA
	QV2M	Specific Humidity at 2 Meters	NASA
	QV10M	Specific Humidity at 10 Meters	NASA
	VPD	Vapor Pressure Deficit	EnvRtype
	SPV	Slope of saturation vapor pressure curve	EnvRtype
	PS	Surface Pressure	NASA
Wind	WS2M	Wind Speed at 2 Meters	NASA
	WS10M	Wind Speed at 10 Meters	NASA
	WD2M	Wind Direction at 2 Meters	NASA
	WD10M	Wind Direction at 10 Meters	NASA
Soil	GWETTOP	Surface Soil Wetness (0-5 cm)	NASA

GWETROOT	Root Zone Soil Wetness (0-100 cm)	NASA
GWETPROF	Profile Soil Wetness	NASA
